# Genomic prediction in hybrid breeding: II. Reciprocal recurrent genomic selection with full-sib and half-sib families

**DOI:** 10.1007/s00122-023-04446-3

**Published:** 2023-08-31

**Authors:** Albrecht E. Melchinger, Matthias Frisch

**Affiliations:** 1 Plant Breeding, TUM School of Life Sciences, Technical University of Munich, 85354 Freising, Germany; 2 Institute of Plant Breeding, Seed Science and Population Genetics, University of Hohenheim, 70599 Stuttgart, Germany; 3 Institute of Agronomy and Plant Breeding II, Justus Liebig University, 35392 Gießen, Germany

## Abstract

**Key message:**

Genomic prediction of GCA effects based on model training with full-sib rather than half-sib families yields higher short- and long-term selection gain in reciprocal recurrent genomic selection for hybrid breeding, if SCA effects are important.

**Abstract:**

Reciprocal recurrent genomic selection (RRGS) is a powerful tool for ensuring sustainable selection progress in hybrid breeding. For training the statistical model, one can use half-sib (HS) or full-sib (FS) families produced by inter-population crosses of candidates from the two parent populations. Our objective was to compare HS-RRGS and FS-RRGS for the cumulative selection gain ($\Sigma \Delta G$), the genetic, GCA and SCA variances ($\sigma_{G}^{2}$,$\sigma_{gca}^{2}$, $\sigma_{sca}^{2}$) of the hybrid population, and prediction accuracy ($r_{gca}$) for GCA effects across cycles. Using SNP data from maize and wheat, we simulated RRGS programs over 10 cycles, each consisting of four sub-cycles with genomic selection of $N_{e} = 20$ out of 950 candidates in each parent population. Scenarios differed for heritability $\left( {h^{2} } \right)$ and the proportion $\tau = 100 \times \sigma_{sca}^{2} :\sigma_{G}^{2}$ of traits, training set (TS) size ($N_{TS}$), and maize vs. wheat. Curves of $\Sigma \Delta G$ over selection cycles showed no crossing of both methods. If $\tau$ was high, $\Sigma \Delta G$ was generally higher for FS-RRGS than HS-RRGS due to higher $r_{gca}$. In contrast, HS-RRGS was superior or on par with FS-RRGS, if $\tau$ or $h^{2}$ and $N_{TS}$ were low. $\Sigma \Delta G$ showed a steeper increase and higher selection limit for scenarios with low $\tau$, high $h^{2}$ and large $N_{TS}$. $\sigma_{gca}^{2}$ and even more so $\sigma_{sca}^{2}$ decreased rapidly over cycles for both methods due to the high selection intensity and the role of the Bulmer effect for reducing $\sigma_{gca}^{2}$. Since the TS for FS-RRGS can additionally be used for hybrid prediction, we recommend this method for achieving simultaneously the two major goals in hybrid breeding: population improvement and cultivar development.

**Supplementary Information:**

The online version contains supplementary material available at 10.1007/s00122-023-04446-3.

## Introduction

Recurrent selection (RS) comprises a multitude of breeding methods sharing as common feature the testing, selection and recombination of genetic units in successive steps of each selection cycle. The basic idea of RS is to improve the population mean by increasing the frequency of favorable alleles over selection cycles without loosing desirable genetic variation for future cycles (Hallauer et al. 2010). While recurrent selection in a broad sense is practiced in every breeding program, scientific studies on the efficiency of RS were generally conducted with closed populations. Quantitative genetics provided the theoretical basis for quantifying the short-term selection gain ($\Delta G$) expected under different RS schemes and the relevant factors contributing to it (Falconer and Mackay 1996; Hallauer et al. 2010). Numerous RS methods have been described in textbooks (e.g., Bernardo 2002, Chap. 9), which differ with regard to the genetic units used in the various steps and whether they deal with intra-population or inter-population improvement.

In hybrid breeding, usually two genetically distant populations are used as base material to take full advantage of hybrid vigor (Melchinger and Gumber 1998). For this reason, RS is mainly concerned with inter-population improvement of both parent populations in reciprocal terms to maximize the mean performance of their hybrid population and the top hybrids between lines developed from the two parent populations (Hallauer et al. 2010). While the technical details of reciprocal recurrent selection can vary depending on the biological requisites of the crop (e.g., multiplication coefficient, ease of pollination control and production of selfed and cross-pollinated seed or doubled-haploid lines), two general categories are distinguished, depending on whether the test units are half-sib (HS) or full-sib (FS) families (Hallauer et al. 2010).

Comstock et al. (1949) proposed HS reciprocal recurrent selection as a method to maximize general combining ability (GCA) in hybrid breeding and mistakenly also mentioned improvement of specific combining ability (SCA). They suggested to select candidates from each population based on their GCA evaluated in HS progenies produced with the opposite population as common tester. Later, the HS method was modified replacing as tester the opposite population by an inbred line or single cross produced from it (Horner et al. 1973).

Full-sib recurrent reciprocal selection was proposed as an alternative to the HS methods (Hallauer 1967; Lonnquist and Williams 1967). The basic idea is to produce and evaluate FS families, corresponding to single crosses (SCs), between pairs of candidates from the two parent populations and recombine within each population the parents (or selfed progenies of them) of crosses with superior hybrid performance to generate the base material for the next breeding cycle. The main advantage of FS over the HS selection is that twice as many candidates can be sampled from each parent population, yielding the same number of SC entries for phenotyping in trials. However, a line with superior GCA is unlikely to be selected in the FS method if crossed by chance to a partner with poor GCA or if their cross has negative SCA. Thus, the positive effect of higher selection intensity is partly offset by a reduced correlation between the selection criterion and GCA, as reflected in the formula for the selection response of the FS method (Bernardo 2002, p. 201).

Genomic selection can readily be integrated into RS methods because the phenotypic data from testing of the genetic units can be used for training the statistical model for genomic prediction. This offers the opportunity to substantially improve the selection response as demonstrated by numerous studies on intra-population improvement in animal and plant breeding (e.g., Crossa et al. 2017; Hickey et al. 2017; Schaeffer 2006). First, the low costs of genotyping permits predicting the breeding value of a large number of non-phenotyped individuals, enabling a tremendous increase in the selection intensity. Second, applying genomic selection for several cycles benefits the selection response by savings in time and costs due to bypassing the time-consuming and expensive step of phenotyping. However, little is known about the persistency of the prediction accuracy and selection gain under different schemes of recurrent genomic selection (Müller et al. 2017). Furthermore, integration of genomic selection into FS reciprocal recurrent selection has so far received little attention in hybrid breeding.

In the present study, we applied fully stochastic forward-in-time simulations based on molecular data from breeding programs in maize and wheat to generate in silico base populations. These were subject to ten cycles of HS-RRGS or FS-RRGS, each cycle consisting of (re-)training and four sub-cycles of genomic selection. Our goal was to (i) investigate the cumulative selection gain ($\Sigma \Delta G$) of the hybrid population over selection (sub-)cycles, (ii) monitor the corresponding changes in the genetic variance ($\sigma_{G}^{2}$) and the GCA and SCA variances ($\sigma_{gca}^{2}$, $\sigma_{sca}^{2}$), and (iii) examine the prediction accuracy ($r_{gca}$) for GCA effects over cycles and sub-cycles. Our main objective was to compare HS-RRGS and FS-RRGS with regard to the short- and long-term selection progress under various scenarios differing in the heritability ($h^{2}$) and proportion $\tau = 100 \times \sigma_{sca}^{2} /\sigma_{G}^{2}$ at the beginning of the selection program as well as the presence or absence of genetically distant parent populations.

## Simulations

### Genetic markers, founder lines and generation of the base populations

Our starting point were two data sets of SNP marker genotypes from maize and wheat. Data set DS1 comprised 145 dent and 111 flint founder lines, serving as female and male lines, respectively, from the maize breeding program of the University of Hohenheim detailed in previous publications (Schrag et al. 2018; Technow et al. 2014; Westhues et al. 2017). Briefly, the lines had been developed either by recurrent selfing for more than six generations or the doubled-haploid (DH) technique and had been selected for per se performance as well as for general combining ability (GCA) of important agronomic traits in testcrosses (TCs) with line testers from the opposite population. All lines had been genotyped with the 50k Illumina SNP chip MaizeSNP50 (Ganal et al. 2011). After a rigorous quality check, a total of 13,813 markers (corresponding to panel $SNP_{all}$) polymorphic in the 256 founder lines were available for our simulations, providing a fairly uniform coverage of the entire maize genome. The genetic map of these SNPs was constructed as detailed by Lanzl et al. (2023) and covered in total 1,442 cM of the maize genome.

Data set DS2 comprised 667 female and 18 male founder lines from spring bread wheat (*Triticum aestivum* L.) used as parents of 1,888 SC hybrids phenotyped in large experiments described by Basnet et al. (2019). The elite parent lines were chosen from CIMMYT’s spring bread wheat program based on per se performance for agriculturally important traits, suitability for producing hybrids and ancestral diversity measured with the co-ancestry coefficient. The female and male parents were genotyped with customary SNP arrays. For our analyses, CIMMYT kindly provided the 10,250 SNP markers (corresponding to panel $SNP_{all}$) underlying the comparison of different methods for genomic prediction of hybrids reported by Basnet et al. (2019). The genetic map of these markers covered in total 3,009 cM of the wheat genome.

For each data set, the base material (denoted as $C_{1,0}$) for the parent populations $F$ (females) and $M$(males) was generated in silico by the following steps: (i) random mate the founder female or male lines, excluding selfing. (ii) Sample 950 S_0_ genotypes from each parent population under the restriction that each founder line contributed no more than 12 gametes to the sample, except for the males in set DS2, where the threshold was raised to a maximum of 55 gametes per founder line due to their small number. (iii) Simulate meiosis in each of the S_0_ genotypes and produce from a randomly chosen gamete one DH line. Thus, we obtained a set of 950 largely unrelated DH lines in both set $F$ and $M$ representing the parent populations at the beginning of genomic selection for both breeding schemes.

### Genetic architecture of the simulated traits

For each data set we followed the same procedure to simulate traits differing in their architecture with regard to the importance of dominance effects detailed in our companion paper (Melchinger et al. 2023). First, a panel $QP$ of 3,000 SNPs, representing an equal proportion of the markers positioned on each chromosome, was randomly sampled from the entire panel $SNP_{all}$ of SNP markers and retained as pool for choosing the QTL positions. The remaining panel $R$ of SNPs in each data set served as markers for genomic prediction. Second, a panel $Q$ of 1,000 QTL randomly chosen from $QP$ were assigned additive ($a_{l}$) and dominance ($d_{l}$) effects as defined in the textbook of Lynch and Walsh (1998). The additive effects $a_{i}$ were drawn from a Gamma distribution with parameter scale = 1.66 and shape = 0.4 following previous studies (Meuwissen et al. 2001; Technow et al. 2012). The dominance effects $d_{l} = a_{l} \times k_{l}$ were obtained multiplying $a_{i}$ by the degree of dominance $k_{l}$ sampled from a normal distribution $k_{l} \sim N\left( {\mu_{k} ,\sigma_{k}^{2} } \right)$. Third, a random panel $Q_{d} \subset Q$ of $n_{d}$ QTL displaying only dominance effects $d_{l}$ was obtained replacing their $a_{l}$ value by zero. Fourth, the additive effects were scaled such that the genetic variance of the hybrid population $F \times M$ in $C_{1,0}$ was $\sigma_{G}^{2}$ = 1. The parameters $\left( {\mu_{k} ,\sigma_{k}^{2} } \right)$ and $n_{d}$ were chosen based on estimates of the degree of dominance and QTL mapping results from numerous studies in maize as detailed in Melchinger et al. (2023) and for wheat, based on a literature survey of various (partially) autogamous crops (see Suppl. Table S1).

For each trait, determined by the location and the genetic effects of the QTL in $Q$, the genotypic value of each candidate from every selection cycle was obtained by summing the respective additive and dominance effects over all QTL. Phenotypic values of TCs (HS-RRGS) or SCs (FS-RRGS) in the training set (TS) of the initial and later selection cycles were obtained by adding to the genotypic values a noise variable from a normal distribution $N\left( {0,\sigma_{e}^{2} } \right)$, where $\sigma_{e}^{2} = \sigma_{G}^{2} \left( {1 - h^{2} } \right)/h^{2}$, $\sigma_{G}^{2}$ is the genetic variance and $h^{2}$ the desired broad-sense heritability of the hybrid population $F \times M$ in $C_{1,0}$.

### Simulation of half-sib and full-sib reciprocal recurrent genomic selection

In our notation, numbers with capital and lower case letters (e.g., $N_{TS}$ and $n_{TS}$) refer to the hybrid and parent populations, respectively. Each selection cycle $C_{t}$ $\left( {t = 1,...,10} \right)$ includes (re-)training the model in sub-cycle $C_{t,0}$ and $s$ $\left( {s = 1,...,4} \right)$ sub-cycles $C_{t,s}$ of genomic selection. The candidates selected in $C_{t,4}$ are used to generate the materials for $C_{t + 1,0}$ by production of DH lines as described below. The two methods HS-RRGS and FS-RRGS differ with respect to (i) the number of line candidates sampled from each population for producing the test units in the TS, (ii) the test units (inter-population HS *versus* FS families) in the TS used for phenotyping and (re-)training the model in $C_{t,0}$, and (iii) the selection criterion (GBLUPs for TC performance in HS-RRGS *versus* GBLUPs for GCA effects in FS-RRGS) used for genomic selection in sub-cycles $C_{t,s}$. However, the two breeding schemes are identical with respect to (a) the identification of superior candidates based on the selection criterion applied by each method, (b) the recombination scheme for generating the material of the next sub-cycle, and (c) the production of DH lines after recombining the candidates selected in $C_{t,4}$ to be used as base material for $C_{t + 1,0}$ (Suppl. Figure S1).

**Fig. 1:**
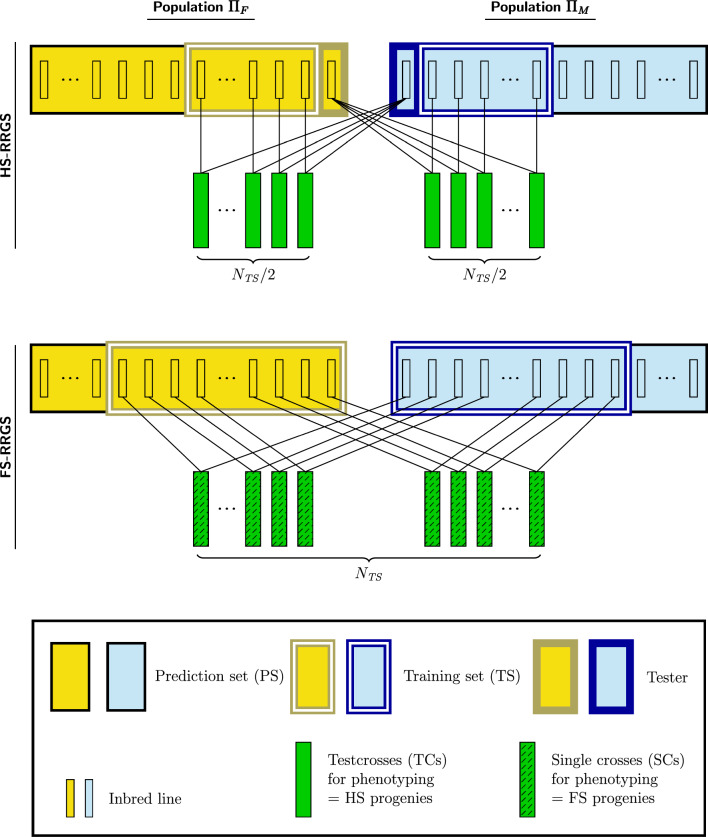
Production of hybrids (green) for the training set using doubled-haploid lines sampled from parent populations Π_*F*_ (females, yellow) and Π_*M*_ (males, blue). Top: Half-sib reciprocal recurrent genomic selection (HS-RRGS) with *N*_*TS*_/2 half-sib families in each population produced by crossing the candidates with an inbred tester from the opposite population. Bottom: Full-sib reciprocal recurrent genomic selection (FS-RRGS) with *N*_*TS*_ single-cross hybrids obtained from paired crosses of DH lines. DH lines in the prediction set of Π_*F*_ and Π_*M*_ are also shown

In each cycle $C_{t}$ of the HS-RRGS scheme, $n_{F}$ female and $n_{M} = n_{F}$ male lines were randomly sampled from parent population $F$ and $M$, respectively, of subcycle $C_{t,0}$ and crossed to a tester from the opposite population (Fig. 1). The TCs from each parent population, adding to a total of $N_{TS} = 2n_{F}$ entries over both parent populations, were phenotyped and served as TS for training the model for TC performance of the respective parent population described below [Eq. (1)]. The GBLUPs of the candidates in sub-cycles $C_{t,s}$ obtained with this model served as selection criterion within cycle $C_{t}$. For each parent population, the tester in cycle $C_{t}$ was the genotype with highest GBLUP value among the 950 DH lines from the opposite population identified in the previous cycle $C_{t - 1,0}$. For cycle $C_{1}$, the tester was the best among 100 randomly chosen DH lines with highest GCA to the opposite population determined by phenotypic evaluation of TCs with the same heritability as in $C_{1,0}$.

In each cycle $C_{t}$ of the FS-RRGS scheme, $n_{F}^{*}$ female and $n_{M}^{*} = n_{F}^{*}$ male lines were randomly sampled from parent population $F$ and $M$ of subcycle $C_{t,0}$ and pairwise crossed (Fig. 1). The $N_{TS} = n_{F}^{*}$ inter-population single-cross (SC) hybrids were phenotyped and served as TS for training the statistical model for hybrid performance described below [Eq. (2)]. The GBLUPs for the GCA of the candidates in $C_{t,s}$, obtained with this model served as selection criterion. Under the assumption of equal expenditures for phenotying the TS for each method, $N_{TS}$ is equal for both methods so that $n_{F}^{*} = n_{M}^{*} = 2n_{F}^{{}} = 2n_{M}^{{}}$, meaning that twice as many lines are sampled from each parent population for (re-)training in FS-RRGS than HS-RRGS.

For both RRGS methods and both parent populations, we applied the same scheme for recombination and recurrent genomic selection in sub-cycles $C_{t,s}$ (Suppl. Figure S1). In $C_{t,0}$, the top $n_{sel,1} = 40$ out of the 950 DH lines were selected from each parent population on the basis of their GBLUPs for TC performance in HS-RRGS and GCA in FS-RRGS, respectively. In each parent population, each selected line was crossed at random with one other selected line. Subsequently, the 20 intra-population single crosses were mated according to a half-diallel design and each of the 190 matings (corresponding to intra-population FS families) contributed exactly five S_0_ progeny to the next generation. The $n = 950$ S_0_ plants constituted the parent population comprising the recombined material of subcycle $C_{t,1}$ and served as starting material for subcycle $C_{t,2}$. They were genotyped in the juvenile stage to select the $n_{sel,2} = 20$ plants with top GBLUPs based on their marker genotype and the statistical model trained in $C_{t,0}$. The $20$ top S_0_ plants were again pairwise mated and five S_0_ progeny plants per intra-population FS family were genotyped for a total of $n = 950$ candidates, comprising the recombined material of subcycle $C_{t,2}$. This procedure was continued up to subcycle $C_{t,4}$, where from each of the $950$ candidates after recombination one DH line was produced in silico to generate 950 DH lines corresponding the material in subcycle $C_{t + 1,0}$. Thus, the allele frequencies in $C_{t,4}$ and $C_{t + 1,0}$ and consequently the mean of the hybrid population between the two parent populations in these sub-cycles are expected to be identical except for sampling effects. Our rationale for selecting $n_{sel,1}$ = 40 DH lines from $C_{t,0}$ but $n_{sel,s}$ = 20 S_0_ plants in advanced subcycles $C_{t,s}$ was to have the same effective population size $N_{e}$ in all sub-cycles.

Our decision to conduct within each cycle four sub-cycles of genomic selection before re-training was based on results examining the persistency of prediction accuracy for genomic selection with synthetic populations (Müller et al. 2017). Our goal was that the prediction accuracy of GBLUPs for the selection criterion should not fall below 25% of the level in $C_{t,0}$.

## Statistical analyses

### Genomic prediction models

For HS-RRGS, we calculated GBLUPs for TC performance of all candidates from each parent population separately and but describe the procedure here only for candidates from population $F$. We used the model,

1 $${\mathbf{y}}_{TC} = {\mathbf{1}}\mu + {\mathbf{Z}}_{F} {\mathbf{u}}_{F} + {\mathbf{e}}$$

where ${\mathbf{y}}_{TC}$ is the vector of TC performance (corresponding to inter-population HS families) of the $n_{F}$ candidates in the TS of population $F$ in sub-cycle $C_{t,0}$, µ is the fixed model intercept, ${\mathbf{Z}}_{F}$ is the design matrix linking the vector ${\mathbf{u}}$ of TC effects of the genotypes from $F$ with ${\mathbf{y}}_{TC}$, ${\mathbf{u}}_{F} \sim N\left( {0,\sigma_{TC,F}^{2} {\mathbf{G}}_{F} } \right)$, where $\sigma_{TC,F}^{2}$ refers to the genetic TC variance of the DH lines from population $F$ in $C_{t,0}$, ${\mathbf{G}}_{F}$ is the genomic relationship matrix among the genotypes in population $F$ calculated as described below, and ${\mathbf{e}}$ is the residual error. The TC performance of all candidates in $F$ was predicted with the formula ${\hat{\mathbf{u}}}_{F} = \sigma_{TC,F}^{2} {\mathbf{G}}_{F,TS} {\mathbf{V}}_{TC,TC}^{ - 1} {\mathbf{y}}_{TC}$, where ${\mathbf{G}}_{F,TS}$ is a sub-matrix of ${\mathbf{G}}_{F}$, referring to the genomic relationship of the genotypes in $F$ and the candidates in the TS, and ${\mathbf{V}}_{TC,TC}^{{}}$ is the phenotypic covariance matrix for TC performance of the candidates in the TS. The variance components required in the above formulas were estimated from the data in the respective TS using the R package regress version 1.3 (Clifford and McCullagh 2006).

For FS-RRGS, we calculated GBLUPs for the GCA of the genotypes in each population based on the following model for inter-population SC hybrids among DH lines (corresponding to inter-population FS families) from the TS

2 $${\mathbf{y}}_{SC} = {\mathbf{1}}\mu + {\mathbf{Z}}_{F} {\mathbf{g}}_{F} + {\mathbf{Z}}_{M} {\mathbf{g}}_{M} + {\mathbf{Z}}_{S} {\mathbf{s}} + {\mathbf{e}},$$

where ${\mathbf{y}}_{SC}$ is the vector of phenotypic values of the $N_{TS} = n_{F}^{*} = 2n_{F}$ SC hybrids in the TS, µ is the fixed model intercept, ${\mathbf{Z}}_{F}$ and ${\mathbf{Z}}_{M}$ are the design matrices linking the random GCA effects of the parent lines from $F$ and $M$, respectively, with their hybrids in the TS, ${\mathbf{Z}}_{S}$ the design matrix of SCA effects for the SC hybrids in the TS. The residuals are again represented by vector ${\mathbf{e}}$. The covariance matrix of the GCA effects ${\mathbf{g}}_{F}$ and ${\mathbf{g}}_{M}$ was ${\mathbf{G}}_{F} \sigma_{gcaF}^{2}$ and ${\mathbf{G}}_{M} \sigma_{gcaM}^{2}$, respectively, and that of SCA effects was ${\mathbf{G}}_{S} \sigma_{sca}^{2}$, where $\sigma_{gcaF}^{2}$, $\sigma_{gcaM}^{2}$ and $\sigma_{sca}^{2}$ are the variance components pertaining to GCA and SCA effects of the complete factorial $F \times M$, ${\mathbf{G}}_{F}$ and ${\mathbf{G}}_{M}$ are the genomic relationship matrices among the genotypes from $F$ and $M$, respectively, and ${\mathbf{G}}_{S} = {\mathbf{G}}_{F} \otimes {\mathbf{G}}_{M}$, with $\otimes$ referring to the Kronecker product. The GCA of all candidates in population $F$ was predicted with the formula ${\hat{\mathbf{g}}}_{F} = \sigma_{gcaF}^{2} {\mathbf{G}}_{F,SC} {\mathbf{V}}_{SC,SC}^{ - 1} {\mathbf{y}}_{SC}$, where ${\mathbf{G}}_{F,SC}$ is the genomic relationship matrix of the individuals in population $F$ and the parents from this population used for producing the SC hybrids in the TS, and ${\mathbf{V}}_{SC,SC}^{{}}$ is the phenotypic covariance matrix of the SC hybrids in the TS. Variance components required for application of GBLUP were obtained from the data in the TS using again the R package regress version 1.3 (Clifford and McCullagh 2006).

### Statistical analyses of the selection (sub-)cycles

With the SNP data from panel $R = SNP_{all} \backslash Q$, we calculated the genomic relationship matrices ${\mathbf{G}}_{F}$ and ${\mathbf{G}}_{M}$ for all genotypes $i$ and $j$ from all sub-cycles of population $F$ and $M$, respectively, according to Schopp et al. (2017), here exemplified for genotypes $i$ and $j$ from population $F$:

3 $${\mathbf{G}}_{F} \left( {i,j} \right) = \frac{{\sum_{l \in R} {\left( {x_{i,l} - p_{l}^{i} } \right)\left( {x_{j,l} - p_{l}^{j} } \right)} }}{{\sqrt {2\sum_{l \in R} {p_{l}^{i} \left( {1 - p_{l}^{i} } \right)} } \sqrt {2\sum_{l \in R} {p_{l}^{j} \left( {1 - p_{l}^{j} } \right)} } }},$$

where $x_{i,l}$, $x_{j,l} \in \left\{ {0,1,2} \right\}$ are the genotypic scores of $i$ and $j$, respectively, reflecting the number of reference alleles in $i$ and $j$, and $p_{l}^{i}$ and $p_{l}^{j}$ are the frequencies of the reference allele at marker $l$ in the sub-cycle(s) of population $F$, from which $i$ and $j$ originated. We also calculated modified Rogers’ distances (Rogers 1972, 1986) on the basis of the markers in panel $R$ between the entire population of 950 DH lines from different sub-cycles $C_{t,0}$ of the same or the opposite parent population.

For both HS-RRGS and FS-RRGS and all sub-cycles $C_{t,s}$, we determined the genotypic values of all hybrids in the factorial between $F$ and $M$ using from each population (a) the 950 DH lines if $s = 0$, (b) the 40 selected DH lines if $s = 1$, or (c) the 40 DH lines corresponding to the parental gametes of the 20 selected S_0_ genotypes if $s > 1$. These provided the basis for calculating with standard procedures (i) the mean $\mu_{F \times M,t,s}$ of all hybrids as well as the GCA and SCA effects and (ii) the genetic variance $\sigma_{G}^{2}$ and variance components $\sigma_{gcaF}^{2}$, $\sigma_{gcaM}^{2}$ and $\sigma_{sca}^{2}$ as well as the proportion $\tau_{t} = 100 \times \sigma_{sca}^{2} :\sigma_{G}^{2}$ for all sub-cycles $C_{t,0}$. The cumulative selection gain $\Sigma \Delta G_{F \times M}$ of the hybrid population $F \times M$ in sub-cycle $C_{t,s}$ was calculated as $\mu_{F \times M,t,s} - \mu_{F \times M,1,0}$ using the fact that $\sigma_{G} = 1$ in $C_{1,0}$.

For each sub-cycle $C_{t,s}$ of HS-RRGS, the prediction accuracy $r_{gca} (TC)$ for GCA effects was calculated by correlating the true GCA values in vectors ${\mathbf{g}}_{F}^{{}}$ and ${\mathbf{g}}_{M}^{{}}$ of the female and male parents of the factorials with their GBLUPs ${\hat{\mathbf{u}}}_{F}^{{}}$ and ${\hat{\mathbf{u}}}_{M}^{{}}$, respectively. Further, we calculated the correlation $r_{{u,\hat{u}}} (TC)$ of the GBLUPs ${\hat{\mathbf{u}}}_{F}^{{}}$ or ${\hat{\mathbf{u}}}_{M}^{{}}$ with their true TC performance ${\mathbf{u}}_{F}^{{}}$ or ${\mathbf{u}}_{M}^{{}}$ determined from the genotypic values of the TCs of the candidates in parent population $F$ and used the same procedure for parent population $M$. For each sub-cycle $C_{t,s}$ of FS-RRGS, the prediction accuracy $r_{gca} \left( {SC} \right)$ for GCA effects was calculated by correlating the true GCA values from the factorials with their GBLUPs ${\hat{\mathbf{g}}}_{F}^{{}}$ or ${\hat{\mathbf{g}}}_{M}^{{}}$ in each sub-cycle $C_{t,s}$. For sub-cycles $C_{t,0}$, these correlations were calculated for the entire set of 950 DH lines and also separately calculated for (i) the DH lines used as parents of the TS and (ii) the DH lines used as parents of the PS. For both methods, $r_{gca}$ values were finally averaged over both parent populations.

With regard to the reduction in the genetic variance due to negative linkage disequilibrium caused by selection, known as the Bulmer effect (Bulmer 1971), we calculated for each sub-cycle $$C_{t,s}$$ also the genic GCA variances ($$\tilde{\sigma }_{gcaF}^{2}$$ and $$\tilde{\sigma }_{gcaM}^{2}$$),the genic SCA variance ($$\tilde{\sigma }_{sca}^{2}$$), and the total genic variance ($$\overset{\lower0.5em\hbox{$\smash{\scriptscriptstyle\frown}$}}{\sigma }_{G}^{2}$$) as follows:

4 $$\begin{gathered} \tilde{\sigma }_{gcaF}^{2} = \sum_{l \in Q} {p_{l}^{F} \left( {1 - p_{l}^{F} } \right)\left[ {a_{l} - \left( {2p_{l}^{M} - 1} \right)d_{l} } \right]}^{2} , \hfill \\ \tilde{\sigma }_{gcaM}^{2} = \sum_{l \in Q} {p_{l}^{M} \left( {1 - p_{l}^{M} } \right)\left[ {a_{l} - \left( {2p_{l}^{F} - 1} \right)d_{l} } \right]}^{2} , \hfill \\ \tilde{\sigma }_{sca}^{2} = 4\sum_{l \in Q} {p_{l}^{F} \left( {1 - p_{l}^{F} } \right)p_{l}^{M} \left( {1 - p_{l}^{M} } \right)d_{l}^{2} } , \hfill \\ \tilde{\sigma }_{G}^{2} = \tilde{\sigma }_{gcaF}^{2} + \tilde{\sigma }_{gcaM}^{2} + \tilde{\sigma }_{sca}^{2} , \hfill \\ \end{gathered}$$

where $p_{l}^{F}$ and $p_{l}^{M}$ is the frequency of the reference allele at QTL $l \in Q$ in sub-cycle $C_{t,s}$ of population $F$ and $M$, respectively, and $a_{l}$ and $d_{l}$ the additive and dominance effect at the QTL. Finally, we computed the ratios $\sigma_{G}^{2} :\tilde{\sigma }_{G}^{2}$,$\left( {\sigma_{gcaF}^{2} + \sigma_{gcaM}^{2} } \right):\left( {\tilde{\sigma }_{gcaF}^{2} + \tilde{\sigma }_{gcaM}^{2} } \right){\text{ and }}\sigma_{sca}^{2} :\tilde{\sigma }_{sca}^{2}$.

For all statistics mentioned above, we calculated the mean and corresponding standard deviations from 400 simulation runs, starting with the production of the parent populations in $C_{1,0}$ from the founder lines in data set DS1 and DS2.

## Results

**Fig. 2:**
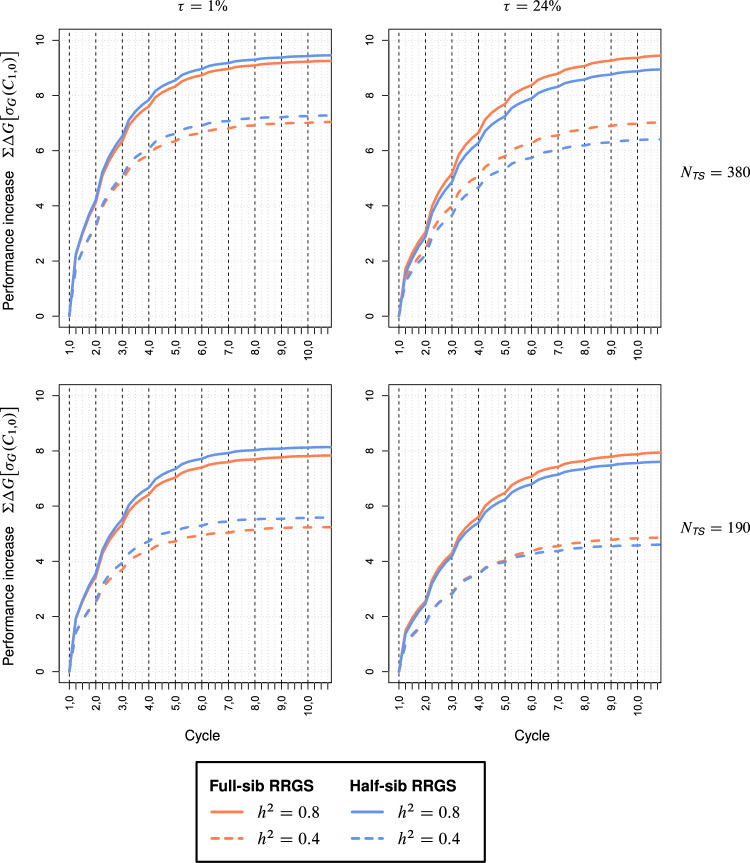
Cumulative selection gain ΣΔ*G* (expressed in units of *σ*_*G*_ in cycle *C*_1,0_) in the hybrid population for full-sib and half-sib reciprocal recurrent genomic selection (RRGS). Results for 10 selection cycles, each consisting of four subcycles, based on SNP data from maize. Scenarios differed for the size *N*_*TS*_ of the training set (TS), heritability *h*^2^, and proportion $\tau = 100\% \times \sigma_{sca}^{2} :\sigma_{G}^{2}$ of the trait

For the maize data set, the curves of the cumulative selection gain $\Sigma \Delta G$ as a function of the (sub-)cycles showed for each scenario a similar pattern for both breeding methods with no intersection between them (Fig. 2). A striking difference existed between the scenarios with high and low $\tau$, where with low $\tau$ the curves for $\Sigma \Delta G$ displayed a much steeper slope in the first cycle and approached faster the selection limit, irrespective of $N_{TS}$. For both values of $\tau$, the selection limit increased by ~ 30% when doubling $h^{2}$ from 0.4 to 0.8, and by ~ 15% when doubling $N_{TS}$ from 190 to 380. For high $\tau$, FS-RRGS had higher $\Sigma \Delta G$ than HS-RRGS with differences being largest (10%) for low $h^{2}$ and $N_{TS} = 380$, intermediate (4–6%) for high $h^{2}$ irrespective of $N_{TS}$, and small (3%) for low $h^{2}$ and $N_{TS} = 190$. For small $\tau$, $\Sigma \Delta G$ for HS-RRGS was ~ 5% greater than for FS-RRGS.

**Fig. 3:**
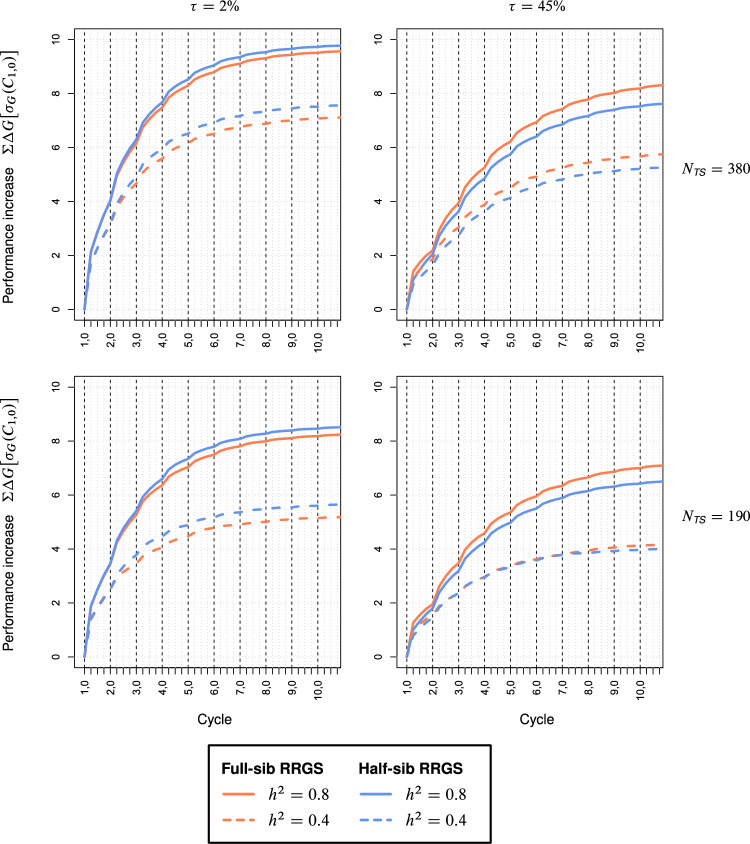
Cumulative selection gain ΣΔ*G* (expressed in units of *σ*_*G*_ in cycle *C*_1,0_) in the hybrid population for full-sib and half-sib reciprocal recurrent genomic selection (RRGS). Results for 10 selection cycles, each consisting of four subcycles, based on SNP data from wheat. Scenarios differed for the size *N*_*TS*_ of the training set (TS), heritability *h*^2^, and proportion $\tau = 100\% \times \sigma_{sca}^{2} :\sigma_{G}^{2}$ of the trait

The curves for $\Sigma \Delta G$ obtained with the wheat data showed essentially the same picture as for maize (Fig. 3). Despite identical assumptions about the genetic architecture of heterotic traits, the contribution of $\sigma_{sca}^{2}$ to the genetic variance $\sigma_{G}^{2}$ among hybrids was much larger in wheat than in maize ($\tau$ = 45% vs. 24%). This was associated with a much slower increase and substantially lower selection limit of $\Sigma \Delta G$ if $\tau$ was high. Furthermore, differences between both methods were more pronounced in that FS-RRGS had 10–12% higher $\Sigma \Delta G$ than HS-RRGS for high $\tau$, whereas HS-RRGS surpassed FS-RRGS by ~ 2 to 10% for all scenarios with small $\tau$.

**Fig. 4:**
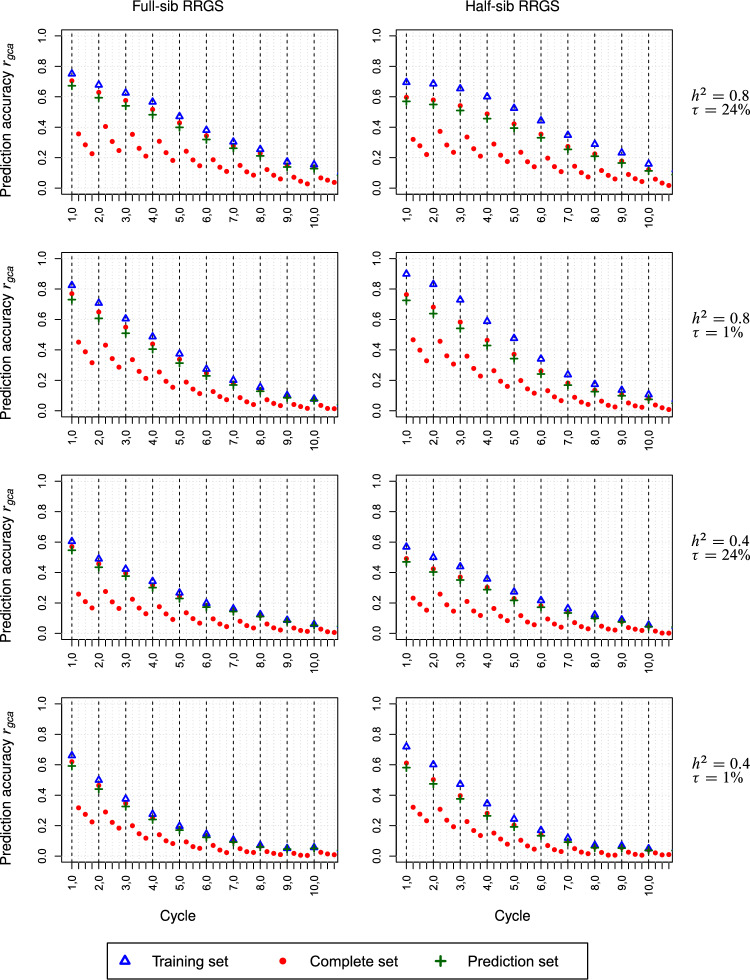
Prediction accuracy *r*_*gca*_ for GCA averaged over populations Π_*F*_ and Π_*M*_ for full-sib and half-sib reciprocal recurrent genomic selection (RRGS). Results for 10 selection cycles, each consisting of four subcycles, based on SNP data from maize. Scenarios differed in the heritability *h*^2^ and the proportion $\tau = 100\% \times \sigma_{sca}^{2} :\sigma_{G}^{2}$ of the trait. The training set size was *N*_*TS*_ = 380

For the maize data set, the reduction of the prediction accuracy $r_{gca}$ as a function of $C_{t,0}$ followed approximately an inverse sigmoid function for high $h^{2}$ and an inverse logarithmic function for low $h^{2}$ (Figs. 4, Suppl. Figure S2). Increasing $h^{2}$ from 0.4 to 0.8 increased $r_{gca}$ by up to 25% for both breeding schemes with only minor modifications due to $\tau$. By comparison, doubling $N_{TS}$ improved $r_{gca}$ by up to 15%. For high $\tau$, in the first cycles $r_{gca}$ was ~ 10% higher for FS-RRGS than HS-RRGS irrespective of $h^{2}$, but this advantage reduced in advanced cycles. For scenarios with low $\tau$, $r_{gca}$ was for both methods at the same level in the first cycle but slightly higher for HS-RRGS in advanced cycles. While $r_{gca}$ for the TS was ~ 15% higher than for the PS in HS-RRGS, differences between both sets were less than 10% for FS-RRGS. Independent of the breeding scheme and scenario, the reduction of $r_{gca}$ in consecutive sub-cycles within a cycle amounted to ~ 40% from sub-cycle $C_{t,0}$ to $C_{t,1}$ but only to ~ 20% in later sub-cycles.

**Fig. 5:**
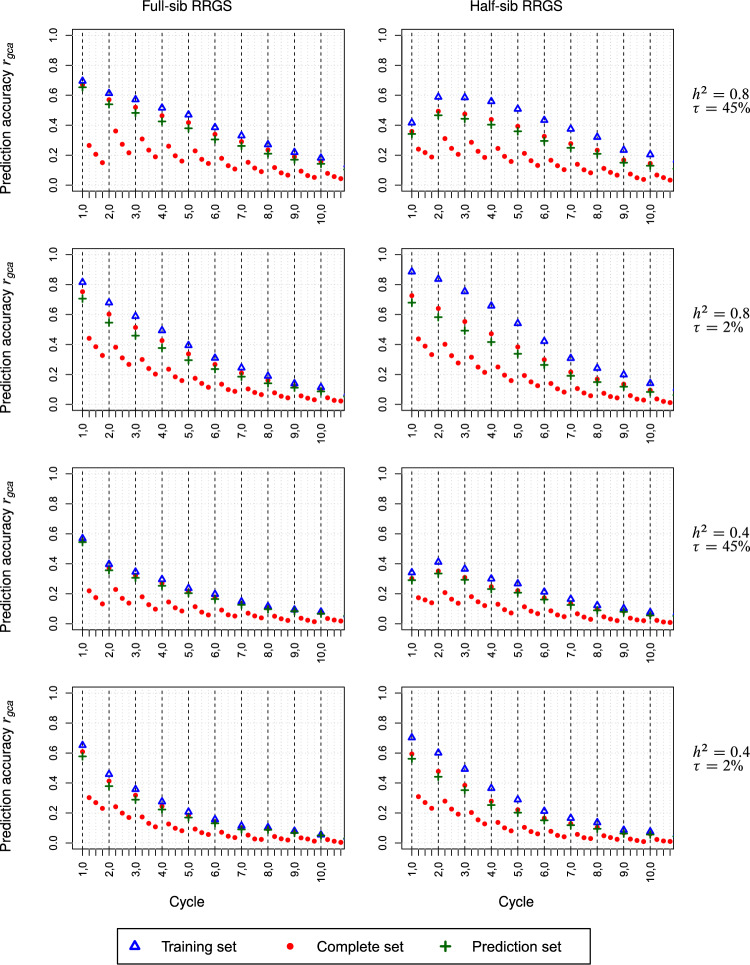
Prediction accuracy *r*_*gca*_ for GCA averaged over populations Π_*F*_ and Π_*M*_ for full-sib and half-sib reciprocal recurrent genomic selection (RRGS). Results for 10 selection cycles, each consisting of four subcycles, based on SNP data from wheat. Scenarios differed in the heritability *h*^2^ and the proportion $\tau = 100\% \times \sigma_{sca}^{2} :\sigma_{G}^{2}$ of the trait. The training set size was *N*_*TS*_ = 380

For the wheat data set, the $r_{gca}$ values for FS-RRGS displayed essentially the same trends as for maize regarding the level and the rate of reduction over (sub-)cycles in all scenarios (Figs. 5, Suppl. Figure S3). For HS-RRGS and scenarios with high $\tau$, $r_{gca}$ started in $C_{1,0}$ at a much lower level yet decreased less in the following sub-cycles and reached in sub-cycles $C_{2,s}$ about the same level as FS-RRGS. For HS-RRGS and scenarios with low $\tau$, $r_{gca}$ was at a similar or higher level for sub-cycles $C_{1,s}$ and $C_{t,s}$ ($t \ge 2$), respectively, and discrepancies between $r_{gca}$ for the TS and PS were much larger than for FS-RRGS.

**Fig. 6:**
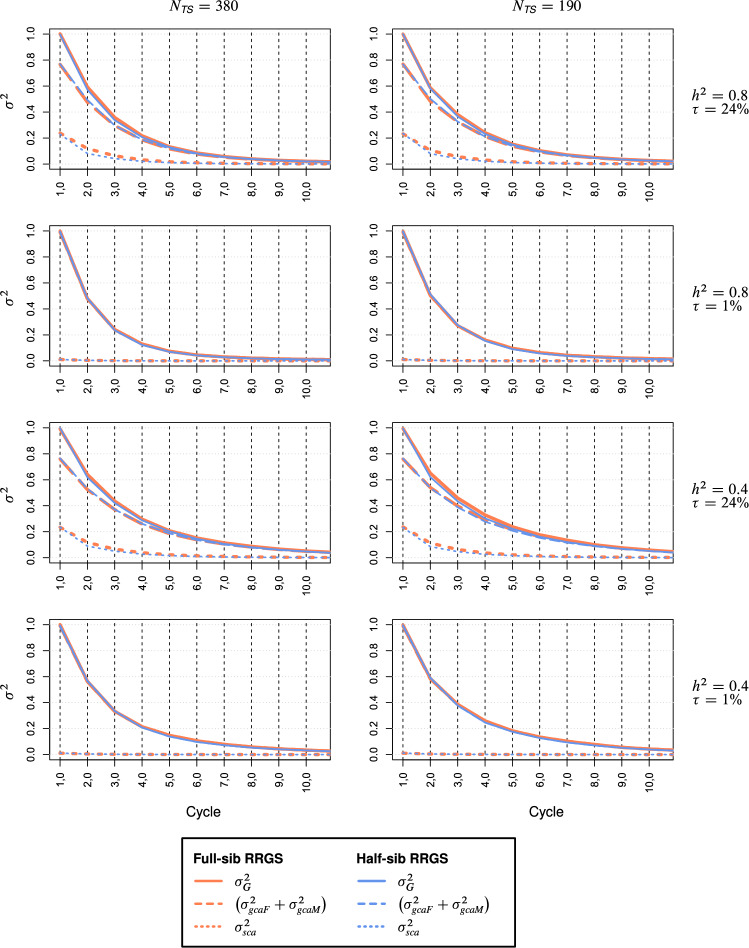
Genetic variances $\sigma_{G}^{2}$, GCA variances $\sigma_{gcaF}^{2} + \sigma_{gcaM}^{2}$, and SCA variances $\sigma_{sca}^{2}$ among hybrids for full-sib and half-sib reciprocal recurrent genomic selection (RRGS). Results for 10 selection cycles based on SNP data from maize. Scenarios differed for the training set size *N*_*TS*_, heritability *h*^2^, and proportion $\tau = 100\% \times \sigma_{sca}^{2} :\sigma_{G}^{2}$ of the trait

For the maize data, the curves for $\sigma_{G}^{2}$ in $C_{t,0}$ were almost identical for both breeding methods (Fig. 6). Lower $h^{2}$ and smaller $N_{TS}$ delayed the reduction in $\sigma_{G}^{2}$ over cycles to a minor extent, whereas low $\tau$ had an accelerating effect. Likewise, the reduction in $\sigma_{gca}^{2} = \sigma_{gcaF}^{2} + \sigma_{gcaM}^{2}$ was almost identical for both breeding methods and followed the same trends as for $\sigma_{G}^{2}$. The only difference was a faster reduction in $\sigma_{sca}^{2}$ for HS-RRGS in $C_{2,0}$ and $C_{3,0}$ for scenarios with high $\tau$. The ratio $\tau_{t} = 100 \times \sigma_{sca}^{2} :\sigma_{G}^{2}$ decreased in an inverse logarithmic function with selection cycles $t$ and the reduction rate was higher for HS-RRGS than FS-RRGS (Suppl. Figure S5).

For the wheat data set, the curves of $\sigma_{G}^{2}$, $\sigma_{gca}^{2}$, and $\sigma_{sca}^{2}$ over selection cycles were at a similar level as for the corresponding scenario in maize (Suppl. Figure S4). For all scenarios with high $\tau$, the rate of reduction in $\sigma_{G}^{2}$ was for both breeding methods slightly higher than in maize due to the high initial value of $\tau$ and the steep reduction in $\sigma_{sca}^{2}$, especially for HS-RRGS. The curves for $\sigma_{gca}^{2}$ were flatter than for maize. For the scenarios with small $\tau$, the reduction in $\sigma_{sca}^{2}$ was congruent for both methods in wheat but remained at higher level than in maize. The proportion $\tau_{t} = 100 \times \sigma_{sca}^{2} :\sigma_{G}^{2}$ dropped substantially during the first cycle with a higher rate for HS-RRGS than FS-RRGS, especially for large $N_{TS}$ (Suppl. Figure S6).

**Fig. 7:**
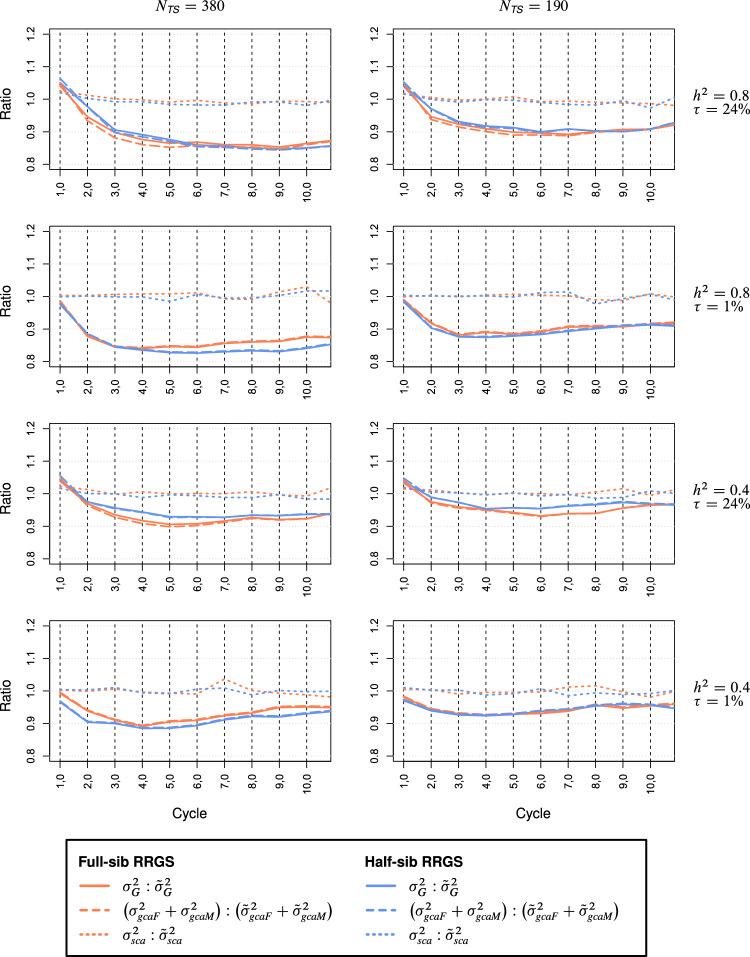
Ratio of genetic to genic variances $\sigma_{G}^{2} :\tilde{\sigma }_{G}^{2}$, of genetic to genic GCA variances $(\sigma_{gcaF}^{2} + \sigma_{gcaM}^{2} ):(\tilde{\sigma }_{gcaF}^{2} + \tilde{\sigma }_{gcaM}^{2} )$, and genetic to genic SCA variances $\sigma_{sca}^{2} :\tilde{\sigma }_{sca}^{2}$ among hybrids for full-sib and half-sib reciprocal recurrent genomic selection (RRGS). Results for 10 selection cycles based on SNP data from maize. Scenarios differed for the training set size *N*_*TS*_, heritability *h*^2^, and proportion $\tau = 100\% \times \sigma_{sca}^{2} :\sigma_{G}^{2}$ of the trait

The curves of $$\sigma_{gca}^{2} :\tilde{\sigma }_{gca}^{2}$$ (ratio of genetic to genic GCA variance) were for the maize and wheat data set largely congruent for both breeding methods (Fig. 7, Suppl. Figure S7). In maize, the ratio dropped from ~ 1.0 at the beginning of selection to values in advanced cycles below 0.9 for $$h^{2}$$ = 0.8 and $$N_{TS}$$ = 380, above 0.9 for $$h^{2}$$ = 0.4 and $$N_{TS}$$ = 190, and close to 0.9 in the other two scenarios. The ratio $$\sigma_{sca}^{2} :\tilde{\sigma }_{sca}^{2}$$ remained for all scenarios close to 1.0. In wheat, the ratio $$\sigma_{gca}^{2} :\overset{\lower0.5em\hbox{$\smash{\scriptscriptstyle\frown}$}}{\sigma }_{gca}^{2}$$ started for scenarios with high $$\tau$$ at a level above 1.6 and approached rapidly its assymptote 1.0, whereas for low $$\tau$$, the ratio was consistently below 1.0 with smaller values for large $$N_{TS}$$. The ratio $$\sigma_{sca}^{2} :\tilde{\sigma }_{sca}^{2}$$ exceeded 1.5 at the beginning and rapidly approached the asymptote 1.0.

## Discussion

Reciprocal recurrent selection of the parent populations is routine in hybrid breeding to support sustainable selection progress. Numerous studies have been conducted in maize and other crops to compare the effectiveness of different methods of reciprocal recurrent selection based on phenotypic data. The comprehensive review by Hallauer et al. (2010) indicates a slightly higher selection gain per cycle for FS over HS reciprocal recurrent selection in accordance with theoretical results (Jones et al. 1971). While these studies were invaluable for experimental quantitative genetics and breeding methodology, they are not meaningful for the comparison of HS-RRGS and FS-RRGS due to the fundamental differences between phenotypic and genomic selection in these breeding methods discussed below. In particularly, it was unknown, whether both methods differ in the prediction accuracy $r_{gca}$ and how this will affect the development of the GCA and SCA variances and the selection gain in the hybrid population over selection cycles. We resorted to simulations for investigating these questions, because they offer a unique flexibility to analyze different scenarios.

### Comparison of reciprocal recurrent selection with half-sib and full-sib families

Reciprocal recurrent selection methods based on phenotypic or genomic data have three steps in common: (i) determination of the selection criterion for all candidates in each parent population, (ii) selection of candidates on the basis of the selection criterion, and (iii) recombination of the selected candidates within each parent population to generate the base material for the next breeding cycle. In phenotypic selection, the selection criterion is based on phenotypic evaluation of HS or FS families produced by inter-population crosses of the candidates in each parent population. By comparison, in genomic selection the selection criterion is calculated on the basis of genomic data of the candidates as input for a statistical model (re-)trained with phenotypic and genomic data of the TS in sub-cycle $C_{t,0}$. With the HS method, the observed or predicted TC performance is used as proxy for the GCA of the candidates under phenotypic and genomic selection, respectively. For phenotypic selection with the FS method, the candidates from each parent population are selected based on the performance of their SC combination with another randomly chosen parent from the opposite population, serving as a proxy for their GCA. Consequently, selection of a candidate depends not only on its own GCA but also on the GCA of its crossing partner and SCA of the hybrid combination. This confounding of effects is reflected in the formula for the selection gain under FS reciprocal recurrent selection, where $\sigma_{sca}^{2}$ and the GCA variance of the opposite population contribute to the denominator (Bernardo 2002, p. 201). The new feature of genomic selection applied to the FS method is that by taking advantage of the genomic relationships among the candidates in the female and male parent populations, it is possible to disentangle the GCA effects of both parents as well as the SCA of the hybrid and predict them individually (cf. our companion paper, Melchinger et al. 2023). This feature of genomic selection enables improving the selection gain in FS-RRGS far beyond that of phenotypic selection with the FS method and has received little attention hitherto. In summary, the methods of HS-RRGS and FS-RRGS compared in our study are identical with regard to steps (ii) and (iii) but they differ with regard to the selection criterion applied in step (i) for all sub-cycles to identify the candidates with highest GCA for subsequent recombination.

### Prediction accuracy for GCA effects and persistency over cycles

Both the HS and FS method have in common that phenotyping of the TCs and SCs, respectively, in the TS does not yield a direct estimate of the GCA of their parental candidates unless—contrary to common practice—a broad-based tester from the opposite population would be used for the HS method. Considering genomic selection as a form of indirect selection, according to the breeder’s equation (Falconer and Mackay 1996, p. 317) the selection gain $\Delta G$ for the target character $Y$ is proportional to the coheritability $r\left( {X,Y} \right) = h_{X} r_{A} \left( {X,Y} \right)$, where $h_{X}$ is the square root of the heritability of the selection criterion $X$, $r_{A} \left( {X,Y} \right)$ is the genetic correlation between $X$ and $Y$, and $r\left( {X,Y} \right)$ is the correlation of $X$ with $Y$, or synonymously the prediction accuracy of $X$ for $Y$. In both HS-RRGS and FS-RRGS, the target character $Y$ is the GCA of the candidates in each parent population with regard to the opposite population. For brevity, we confine our further discussion to the GCA of candidates from population $F$. For HS-RRGS, $X$ is the predicted TC performance of the female candidates in combination with the tester from the male population on the basis of Eq. (1). Thus, $h_{X} = r_{{u,\hat{u}}} \left( {TC} \right)$ is the prediction accuracy of the GBLUPs for TC performance and $r_{A} = r_{g} (TC,gca)$ is the genetic correlation of TC performance with GCA. If the GCA of candidates and their SCA effects with the tester are uncorrelated, which holds strictly true for a randomly chosen tester but approximately also for testers selected for high or low GCA (results not shown), we can express $r_{g} (TC,gca)$ as a function of $\sigma_{gcaF}^{2}$ and $\sigma_{sca}^{2}$

5 $$r_{g} \left( {TC,gca} \right) = \sqrt {\frac{{\sigma_{gcaF}^{2} }}{{\sigma_{gcaF}^{2} + \phi \sigma_{sca}^{2} }}} = \sqrt {\frac{1 - \tau }{{1 + \tau \left( {2\phi - 1} \right)}}} ,$$

where $\phi$ depends on the tester, with $\phi = 1$ for an inbred line, $\phi = 1/2$ for a SC tester from two unrelated lines, and $\phi = 0$ if the entire opposite population is used as tester. Thus, $r_{g} \left( {TC,gca} \right)$ can be much smaller than 1.0 (e.g., 0.77 and 0.62 for an inbred tester, if $\tau = 24\%$ or 45%, respectively). Summarizing, the prediction accuracy $r_{gca} \left( {TC} \right)$ for GCA effects in HS-RRGS is obtained as product

6 $$r_{gca} \left( {TC} \right) = r_{{u,\hat{u}}} \left( {TC} \right)r_{g} \left( {TC,gca} \right)$$

Since $r_{{u,\hat{u}}} \left( {TC} \right)$ is little affected by the size of $\tau$(results no shown), this formula explains why the values for $r_{gca} \left( {TC} \right)$ differed in $C_{1,0}$ only by a constant factor between traits with high and low values of $\tau$ (Suppl. Figure S8).

In FS-RRGS, the GCA of the candidates is directly predicted on the basis of the SC performance of the hybrids in the TS. Thus, $h_{X} = r_{{u,\hat{u}}} \left( {SC,gca} \right)$ is the prediction accuracy of the GBLUPs for the GCA effects obtained from the statistical model in Eq. (2) and $r_{A} = r_{g} \left( {gca,gca} \right) = 1$ so that $r_{gca} (SC) = r_{{u,\hat{u}}} (SC,gca)$. We disregarded SCA effects in the calculation of GBLUPs for GCA effects in Eq. (2), which would require reliable estimates of $\sigma_{sca}^{2}$ that are difficult to obtain in applied breeding programs. Altogether, the higher $r_{gca}$ values for FS-RRGS than HS-RRGS in our simulations are in harmony with a recent experimental study showing for forage traits in maize a higher prediction accuracy for sparse factorial designs than TC designs (Lorenzi et al. 2022).

The persistency of $r_{gca}$ over sub-cycles is of crucial importance for the decision, after how many sub-cycles re-training becomes necessary. The decline of $r_{gca}$ in successive sub-cycles followed generally the same pattern for FS-RRGS and HS-RRGS (Figs. 4, 5, Suppl. Figure S2 and S3), albeit at slightly different levels which depended mainly on the $r_{gca}$ values in $C_{t,0}$ discussed above. The substantial drop of ~ 50% from $C_{t,0}$ to $C_{t,1}$ and the much smaller reductions in subsequent sub-cycles are in harmony with the simulation results of Müller et al. (2017) for genomic prediction of TC performance in synthetics produced from a large number of parents. These authors attributed this pattern to the fact that in sub-cycle $C_{t,0}$, pedigree relationships between candidates in the TS and PS captured by markers are the main driver of $r_{gca}$. However, the variation in pedigree relationships between TS and PS erodes in advanced sub-cycles so that linkage disequilibrium (LD) between SNPs and QTL becomes the primary source for genomic prediction, which is more persistent due to the low recombination rate between adjacent loci.

The reduction in $r_{gca}$ across cycles occurred at a much lower rate than expected from theory (Daetwyler et al. 2008) based on the decline in $h^{2}$ calculated from the values for $\sigma_{gca}^{2}$ (Figs. 4, 6, Suppl. Figure S2 and S3) and constant $\sigma_{e}^{2}$ in all cycles. This is most likely attributable to the accumulation of negative LD between adjacent loci over (sub-)cycles as a result of the Bulmer effect discussed below. Thus, LD became in advanced cycles a more important driver of genomic prediction and this hypothesis is supported by the relatively smaller gap in $r_{gca}$ between $C_{t,0}$ and $C_{t,1}$ for higher values of *t*. A notable exception from this trend was the increase in $r_{gca}$ between $C_{1,0}$ and $C_{2,0}$ in HS-RRGS of wheat for $\tau = 45\%$. This was accompanied by a substantial reduction in $\sigma_{sca}^{2}$, much stronger than for $\sigma_{gca}^{2}$ (Suppl. Figure S4 and S6), which explains the increased prediction accuracy of GCA effects in the subsequent cycle.

We decided to re-train the model after four sub-cycles because at this stage, $r_{gca}$ had in both breeding schemes and all scenarios dropped to a level, where it seems no longer rewarding to continue genomic selection. In practice, the decision for re-training aims at maximizing the selection gain per time unit under given financial and logistic resources. Hence, it depends on numerous factors detailed by Müller et al. (2017), which are beyond the scope of this paper.

Combining the TS data from cycle $t - 1$ to the TS for re-training the model in cycle $t$ hardly improved the prediction accuracy of both methods (results not shown). Since successive cycles were separated by four generations of selection and intermating, they display low levels of pedigree relationships and linkage phase similarity so that combining old and new sources of information had little effect on the prediction accuracy. For HS-RRGS, the carry-over of phenotypic information across selection cycles was further hampered by choosing in each cycle a new tester with outstanding TC performance in the previous cycle.

### Development of GCA and SCA variances over selection cycles

Since $r_{gca}$ differed little between HS-RRGS and FS-RRGS and the selection and recombination schemes were identical for both methods (Suppl. Figure S1), the curves for $\sigma_{G}^{2}$ (and its major component $\sigma_{gca}^{2}$) as a function of the selection cycle were almost congruent for both breeding methods (Fig. 6, Suppl. Figure S4). Based on the effective population size $N_{e} = 20$ of the selected fraction in all sub-cycles, the expected reduction in $\sigma_{gca}^{2}$ due to genetic drift in one cycle (≙ four sub-cycles) amounts to $1-\left( {0.975} \right)^{4} \overset{\wedge}{=}10\%$ (Falconer and Mackay 1996, p. 59). This corresponds to about one quarter of the ~ 40% drop in $\sigma_{gca}^{2}$ observed from $C_{1,0}$ to $C_{2,0}$ in all scenarios.

The remaining larger part of the reduction in $\sigma_{gca}^{2}$ is due to selection and reflects the changes in allele frequencies and build-up of negative covariances among QTL due to the Bulmer effect (Bulmer 1971). To disentangle these causes, we examined the reduction in the genic GCA variance $\tilde{\sigma }_{gca}^{2}$, which reflects exclusively the changes in allele frequencies due to drift and selection, including hitchhiking effects, but which is unaffected by the Bulmer effect. Since the QTL effects were randomly assigned to the QTL in set $Q$, $\sigma_{gca}^{2}$ and $\tilde{\sigma }_{gca}^{2}$ were similar in cycle $C_{1,0}$ as expected (Fig. 7, Suppl. Figure S7). An exception was the wheat data set, where the ratio of genetic to genic variance was much larger than 1.0 for all variance components, most likely due to the high level of LD among adjacent loci in the population of males and its high linkage phase similarity with the population of females. After two selection cycles, however, $\sigma_{gca}^{2}$ was always about 10% smaller than its counterpart $\tilde{\sigma }_{gca}^{2}$ due to the predominance of negative covariances between pairs of QTL, revealing that the Bulmer effect explains a substantial proportion of the reduction in $\sigma_{gca}^{2}$ in all scenarios of both breeding schemes. The increase in $\sigma_{gca}^{2}$ from cycle $C_{1,0}$ to $C_{2,0}$ for $\tau = 45\%$ in the wheat data set (Suppl. Figure S4) is attributable to the substantial increase of the genetic distance between the parent populations (results not shown) as a consequence of selection, which entails an increase of $\sigma_{gca}^{2}$ at the expense of a sharp reduction in $\sigma_{sca}^{2}$, as expected from theory (Reif et al. 2007).

The higher rate of reduction in $\sigma_{sca}^{2}$ than $\sigma_{gca}^{2}$ over selection cycles for scenarios with high $\tau$ (Fig. 6, Suppl. Figure S4) observed for both methods is partly attributable to the stronger effect of genetic drift on the reduction in $\sigma_{sca}^{2}$ because the latter depends on the product of the variance-reducing effect of inbreeding in each parent population and amounts to $\left( {1 - \left( {0.975} \right)^{4} } \right)^{2} \overset{\wedge}{=}19\%$ over four sub-cycles. Interestingly, the ratio $\sigma_{sca}^{2} :\tilde{\sigma }_{sca}^{2}$ was close to 1.0 across all selection cycles in maize and after four cycles in wheat suggesting that covariances of dominance effects display no Bulmer effect. The slower reduction of $\sigma_{sca}^{2}$ for FS-RRGS compared to HS-RRGS can be explained by differences in the selection pressure for SCA effects. Since the TS for FS-RRGS is composed of a large number of inter-population SCs, each parent population acts as a kind of broad-based tester for the opposite population so that the predicted GCA effects are hardly affected by SCA effects. By contrast, GCA and SCA are confounded in the TC performance for HS-RRGS so that selection affects equally both types of effects.

Altogether, our simulation results support the apprehension of previous studies (e.g., Heslot et al. 2015) that the high selection intensity enabled by genomic selection can cause a rapid depletion of $\sigma_{G}^{2}$ in both breeding schemes. The rate of reduction in GCA variances is closely associated with the level of $r_{gca}$ and consequently, the risk of narrowing the genetic base is greatest under those conditions, where genomic selection is most successful. Hence, it seems prudent to estimate regularly the genetic variance components in the TS so that breeders can take effective counter measures to avoid a genetic narrowing of their germplasm.

### Selection gain and limits under both breeding methods

We determined the cumulative selection gain $\Sigma \Delta G$ achieved in the hybrid population by the mean of the genotypic values of the complete factorial of crosses among the gametes produced by the selected candidates. This corresponds to the sum of the selection gain for GCA in the two parent populations except for marginal deviations due to dominance effects. Thus, differences in the curves of $\Sigma \Delta G$ between both methods are primarily due to the corresponding differences in $r_{gca}$ and GCA variances. A further factor was that the TS included twice as many candidates for FS-RRGS as for HS-RRGS because they had slightly higher $r_{gca}$ than the candidates in the PS (Figs. 4, 5, Suppl. Figure S2 and S3), their contribution to ΔG in $C_{t,0}$ was slightly higher.

We observed no crossing of the curves of $\Sigma \Delta G$ for HS-RRGS and FS-RRGS (Figs. 2, 3). Hence, the superiority of one method observed in the first cycle carries over to subsequent cycles. Thus, only few sub-cycles are required for comparing both methods in breeding experiments. Moreover, there is no incentive for changing the breeding method between early and late cycles. Only if no genetically distant parent populations are available in the initial phase of the program as applied to our example from wheat, one might start with FS-RRGS due to its higher $r_{gca}$ in $C_{1,0}$ and possibly continue afterwards with HS-RRGS depending on the genetic architecture of the trait(s) of primary interest.

For both methods, the rate of increase in $\Sigma \Delta G$ and the selection limit were much higher for traits with low $\tau$. In this case, half of the upper limit for $\Sigma \Delta G$ was already reached after one cycle (≙ four sub-cycles), whereas for large $\tau$ at least twice as many cycles were required. Doubling $h^{2}$ increased $\Sigma \Delta G$ more than doubling $N_{TS}$ from 190 to 380. Hence, to ensure a high long-term $\Sigma \Delta G$, it is particularly important to use large $N_{TS}$ in combination with high $h^{2}$ at the beginning of RRGS because otherwise numerous favorable alleles are lost, which cannot be compensated by increased inputs in later cycles.

Within all cycles, the selection gain was by far largest in sub-cycle $C_{t,0}$ (Figs. 2, 3). This is attributable to the strong reduction in $r_{gca}$ and GCA variances in later sub-cycles discussed above. Further, selection in $C_{t,0}$ was among DH lines with the full amount of $\sigma_{gca}^{2}$ whereas selection in later sub-cycles was among S_0_ plants with half the amount of $\sigma_{gca}^{2}$.

An important question for $\Sigma \Delta G$ in HS-RRGS concerns the choice of the tester. According to theory (Rawlings and Thompson 1962) and experimental results, low-performing testers with low frequency of favorable alleles at important loci are most effective for improving GCA (Hallauer et al. 2010). By contrast, we adapted the common practice in hybrid breeding and chose from the previous cycle the top line with highest predicted GCA from the opposite population as tester, because in this case the best TCs evaluated in the TS represent already promising hybrids of direct use for commercialization. For large $\tau$, it follows from Eq. (5) that using a single-cross tester increases prediction accuracy for GCA effects and consequently also $\Sigma \Delta G$, but this would rule out direct development. Updating and choice of the best tester from the lines of the previous cycle most likely helped to push the genetic composition of each parent population in a direction that it optimally complemented the opposite population of the heterotic pattern, but further research is warranted to investigate the choice of the tester on the long-term selection in HS-RRGS.

In our selection scheme, we restricted the maximum number of individuals selected from an intra-population FS family in the recombination step to five genotypes. Alternatively, inbreeding in each parent population could be mitigated by applying optimum contribution selection (Woolliams et al. 2015) and/or selection amongst heterozygous genotypes with greater gametic variance (Bijma et al. 2020; Lehermeier et al. 2017; Müller et al. 2018), which could be further combined with optimal cross selection (Gorjanc et al. 2018). These modifications could be applied equally to both methods but most likely do not change their ranking, yet further research is warranted to quantify their effect.

As a spin-off of reciprocal recurrent selection, breeders are interested to identify in every (sub-)cycle the best genotypes for cultivar development. The usefulness criterion proposed by Schnell and Utz (1975) is a suitable tool for comparing the two methods with respect to the expected performance level of the selected genotypes. For hybrid genotypes, usefulness is a function of the mean of the hybrid population and $\sigma_{gca}^{2}$ of each parent population. Since the two methods hardly differed in $\sigma_{gca}^{2}$ for most scenarios, their ranking with regard to the usefulness criterion followed closely that for $\Sigma \Delta G$ discussed above. With FS-RRGS, however, it is possible to predict not only the GCA but also the performance of the hybrids in the entire factorial between all lines (i.e., TS ∪ PS) in the parent populations, which opens new avenues for improving the efficiency of hybrid breeding.

### Limitations of our study

While simulations are a powerful tool for analyzing the questions addressed in our study, they are meaningful only if the underlying model allows a simplified but nevertheless faithful representation of reality. This is of utmost importance for long-term selection because even minor deviations irrelevant for a single selection cycle will accumulate exponentially over cycles and may therefore lead to erroneous conclusions regarding the long-term prospects. A critical assumption underlying our simulations concerned the genetic architecture of the traits. We choose our genetic models ignoring epistasis based on experimental data of maize detailed in our companion paper (Melchinger et al. 2023). Since little comparable information is available on self-fertilizing species, we resorted to a literature review about the importance of GCA and SCA variances in autogamous and partially allogamous crops (Suppl. Table S1). In general, the $\tau$ values underlying our simulations were in agreement with the estimates from these experimental studies.

The marker densities in our simulations correspond to those of custom-made SNP chips currently used in the commercial sector. Higher marker densities resulted only in marginal improvements of $r_{gca}$ (DoVale et al. 2022; Müller et al. 2017). We ignored the effects of mutation for retarding the depletion of $\sigma_{G}^{2}$ (Walsh and Lynch 2018) as this should affect the long-term $\Sigma \Delta G$ of both methods equally. We also disregarded genotype × environment interactions in the phenotyping of the TS. If a highly selected tester with proven stability over environments is used in HS-RRGS, one might expect that TCs show smaller interactions and consequently higher $h^{2}$ than the unselected hybrids in the TS for FS-RRGS, which would increase their $r_{gca}$. Experimental comparisons of both methods should therefore be conducted in multiple environments and consider genotype × environment interactions in the statistical model for analyses due to their impact on the prediction accuracy (Acosta-Pech et al. 2017; Basnet et al. 2019; Crossa et al. 2017).

## Conclusions

For hybrid breeding in crops and heterotic patterns with high $\sigma_{sca}^{2}$ for important agronomic traits such as yield in temperate maize, FS-RRGS is the method of choice because with high $h^{2}$ or large $N_{TS}$, $\Sigma \Delta G$ is ~ 3–10% higher than what is achieved with HS-RRGS. In contrast, for traits with mainly additive gene action, one might consider HS-RRGS, because it was always on par or slightly better than FS-RRGS when $\sigma_{sca}^{2}$ was low although differences were generally small. In view of the daunting reduction in the genetic variances at risk with the rapid cycles and high selection intensity in genomic selection, the benefits of optimum contribution selection and optimum cross selection should be investigated. In addition, the effects of additional generations of recombination before model re-training and various intermating schemes of the selected candidates on the short- and long-term selection gain deserves further attention. Altogether, implementing FS-RRGS promises tremendous progress in hybrid breeding by integrating reciprocal recurrent genomic selection with genomic prediction of hybrid performance for cultivar development.

## Supplementary Information

Below is the link to the electronic supplementary material.

Supplementary file1 (PDF 1224 KB)

## Data Availability

The molecular data of the 246 maize inbreds from data set DS1 and all code used in the article can be found at Github (https://github.com/PopGen-Giessen/MelchingerFrisch2023). The molecular data of the 685 wheat lines from data set DS2 can be requested from CIMMYT.
