# Supplementary material for: Genomic prediction in hybrid breeding: II. Reciprocal recurrent genomic selection with full-sib and half-sib families
Source: Theor Appl Genet. 2023 Aug 31;136(9):203. doi: 10.1007/s00122-023-04446-3 (PMC10471712; doi:10.1007/s00122-023-04446-3)
Supplement: Supplementary file 1 — Supplementary file1 (PDF 1224 KB) [file 122_2023_4446_MOESM1_ESM.pdf]

Genomic prediction in hybrid breeding: II. Reciprocal recurrent genomic selection with full-sib and half-sib families

Albrecht E. Melchinger and Matthias Frisch

Supplementary Tables and Figures

Table S1: Survey for estimates of general combining ability (GCA, σ^2_{gca}) and specific combining ability (SCA, σ^2_{sca}) variances for hybrids of (partially-)autogamous species for important agronomic traits reported in the literature.

Species	Trait	σ^2_{gca} †	σ^2_{sca} ‡	τ	Source
Barley	Yield	0.36	0.04	10.0%	Philipp et al. (2016)
Rapeseed	Yield	546720	14833	2.7%	Hu et al. (2021)
Rice	Yield	0.430	0.347	43.0%	Beukert et al. (2017)
	Yield	14.81	11.79	44.3%	Gramaje et al. (2020)
	Plant height	0.61	8.44	93.3%	Gramaje et al. (2020)
Triticale	Yield	63.85	1.28	2.0%	Oettler et al. (2003)
	Plant height	58.23	0.07	1.2%	Oettler et al. (2003)
Wheat	Yield	0.058	0.028	32.6%	Longin et al. (2013)
	Plant height	17.86	1.16	6.1%	Longin et al. (2013)
	Heading date	2.05	0.05	2.4%	Longin et al. (2013)
	Yield	0.200	0.082	29.1%	Gowda et al. (2012)
	Yield	0.03	0.02	50.0%	Zhao et al. (2015)

† Sum of σ^2_{gcaF} and σ^2_{gcaM} if presented separately in the reference

‡ $\tau = 100\% \times \frac{\sigma^2_{sca}}{\sigma^2_{gcaF} + \sigma^2_{gcaM} + \sigma^2_{sca}}$ is the proportion of SCA variance in the total genetic variance among hybrids

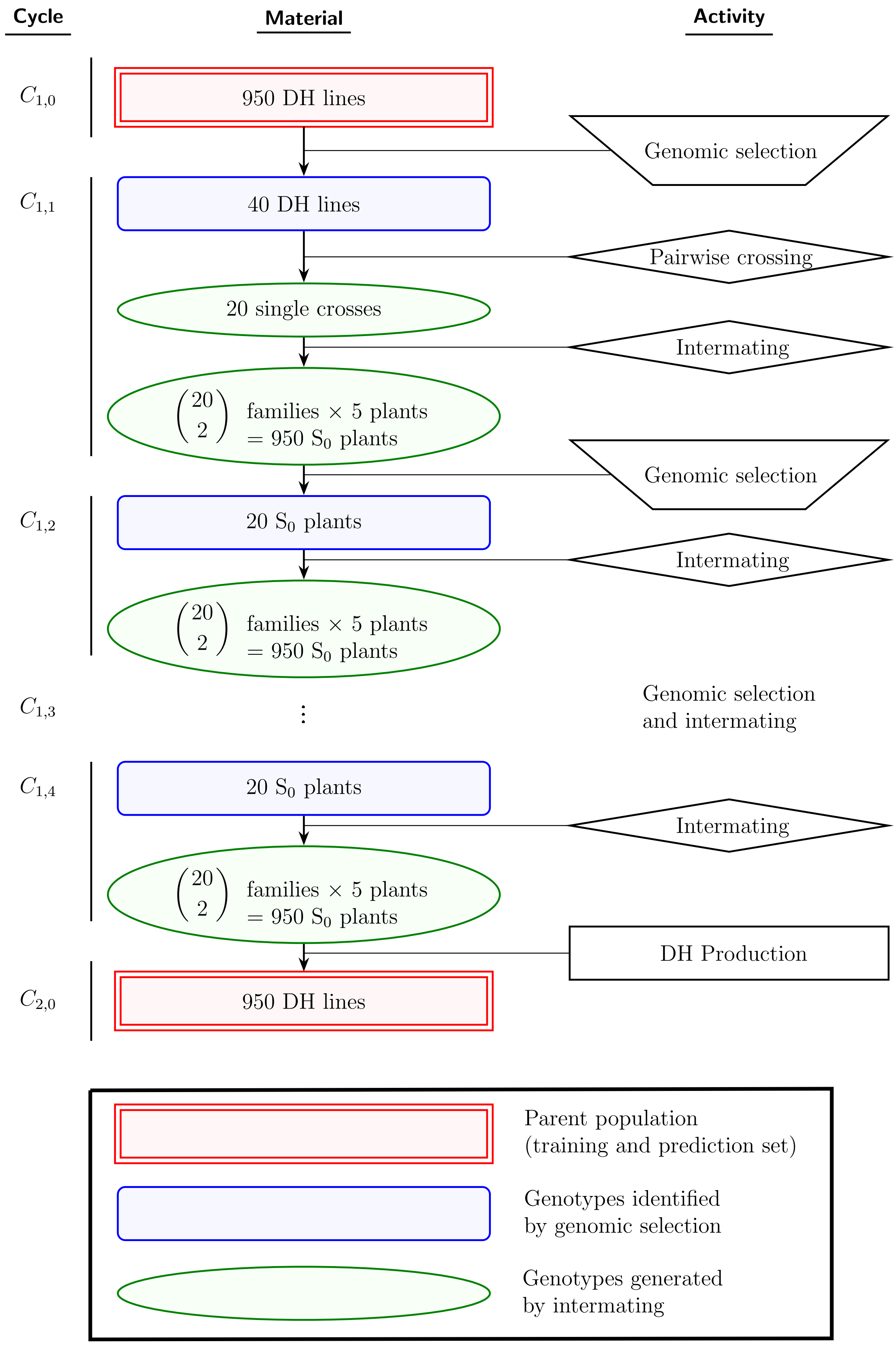

Figure S1: Schematic representation of selection cycle C_1 with (i) model training and genomic selection ($C_{1,0}$), (ii) subsequent subcycles $C_{1,1}$ to $C_{1,4}$ of genomic selection, and (iii) generating in sub-cycle $C_{1,4}$ the material used for re-training the model in cylce $C_{2,0}$.

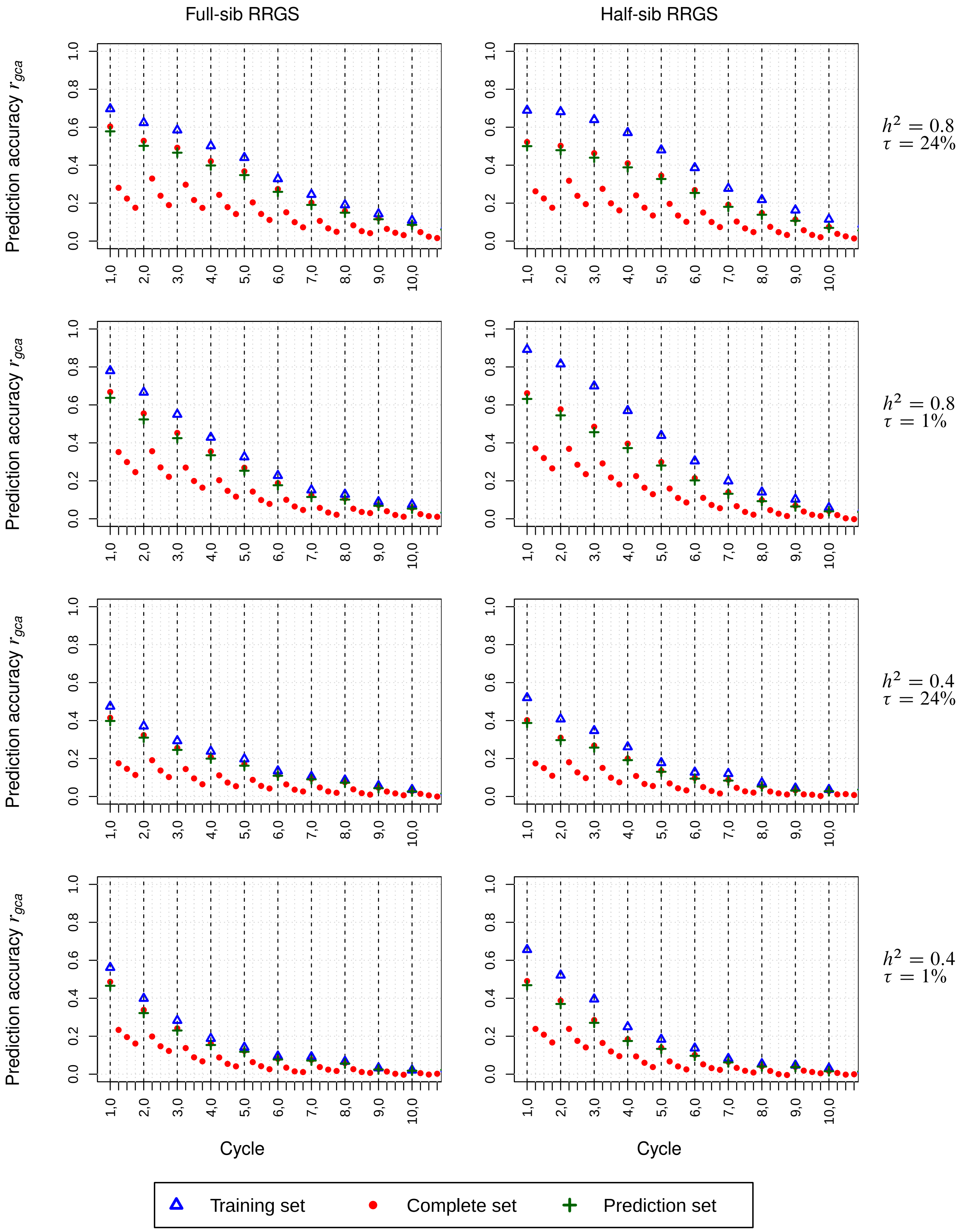

Figure S2: Prediction accuracy r_{gca} for GCA averaged over populations Π_F and Π_M for full-sib and half-sib reciprocal recurrent genomic selection (RRGS). Results for 10 selection cycles, each consisting of four subcycles, based on SNP data from maize. Scenarios differed in the heritability h^2 and the proportion $\tau = 100\% \times \sigma^2_{sca} : \sigma^2_G$ of the trait. The training set size was $N_{TS} = 190$.

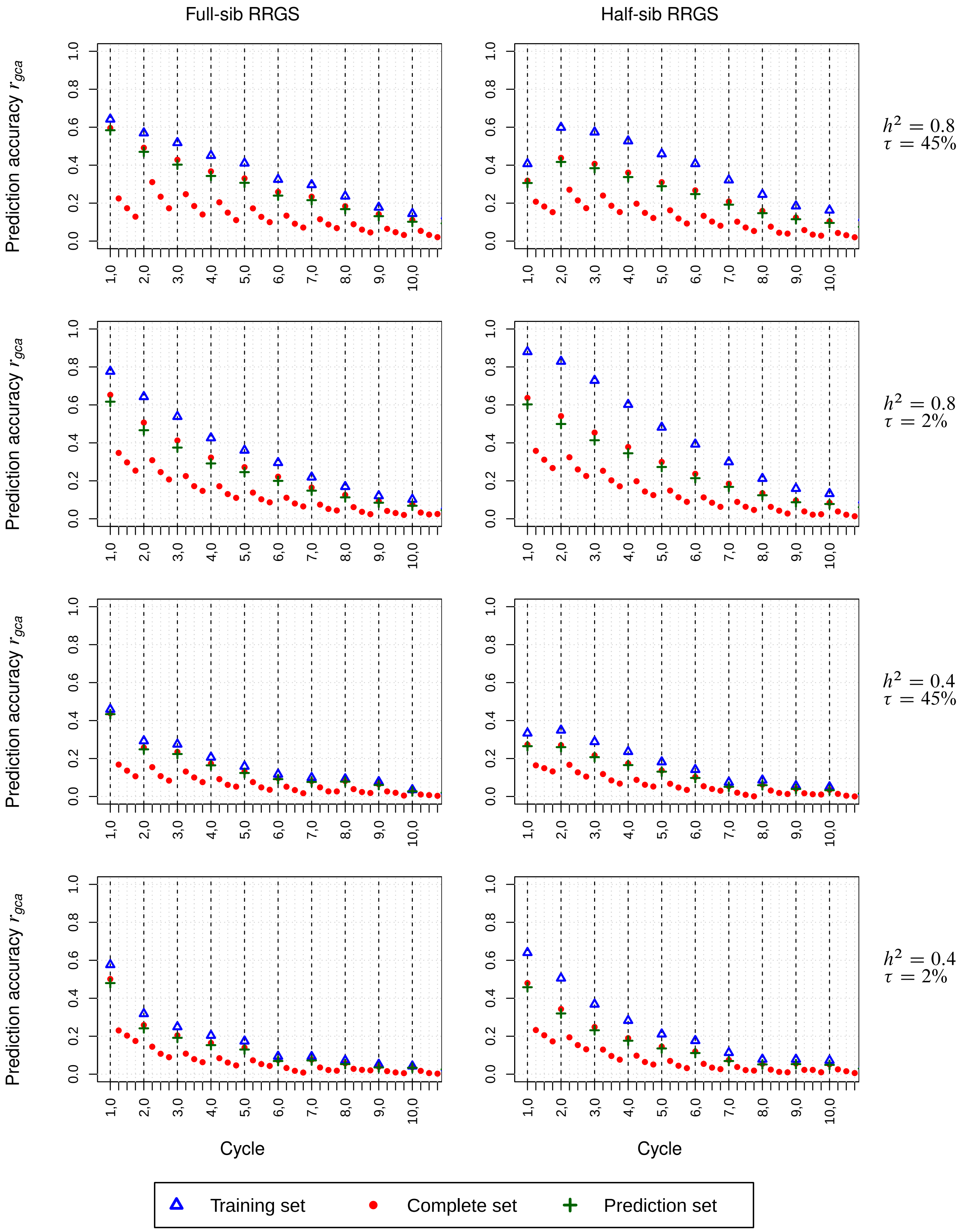

Figure S3: Prediction accuracy r_{gca} for GCA averaged over populations Π_F and Π_M for full-sib and half-sib reciprocal recurrent genomic selection (RRGS). Results for 10 selection cycles, each consisting of four subcycles, based on SNP data from wheat. Scenarios differed in the heritability h^2 and the proportion $\tau = 100\% \times \sigma^2_{sca} : \sigma^2_G$ of the trait. The training set size was $N_{TS} = 190$.

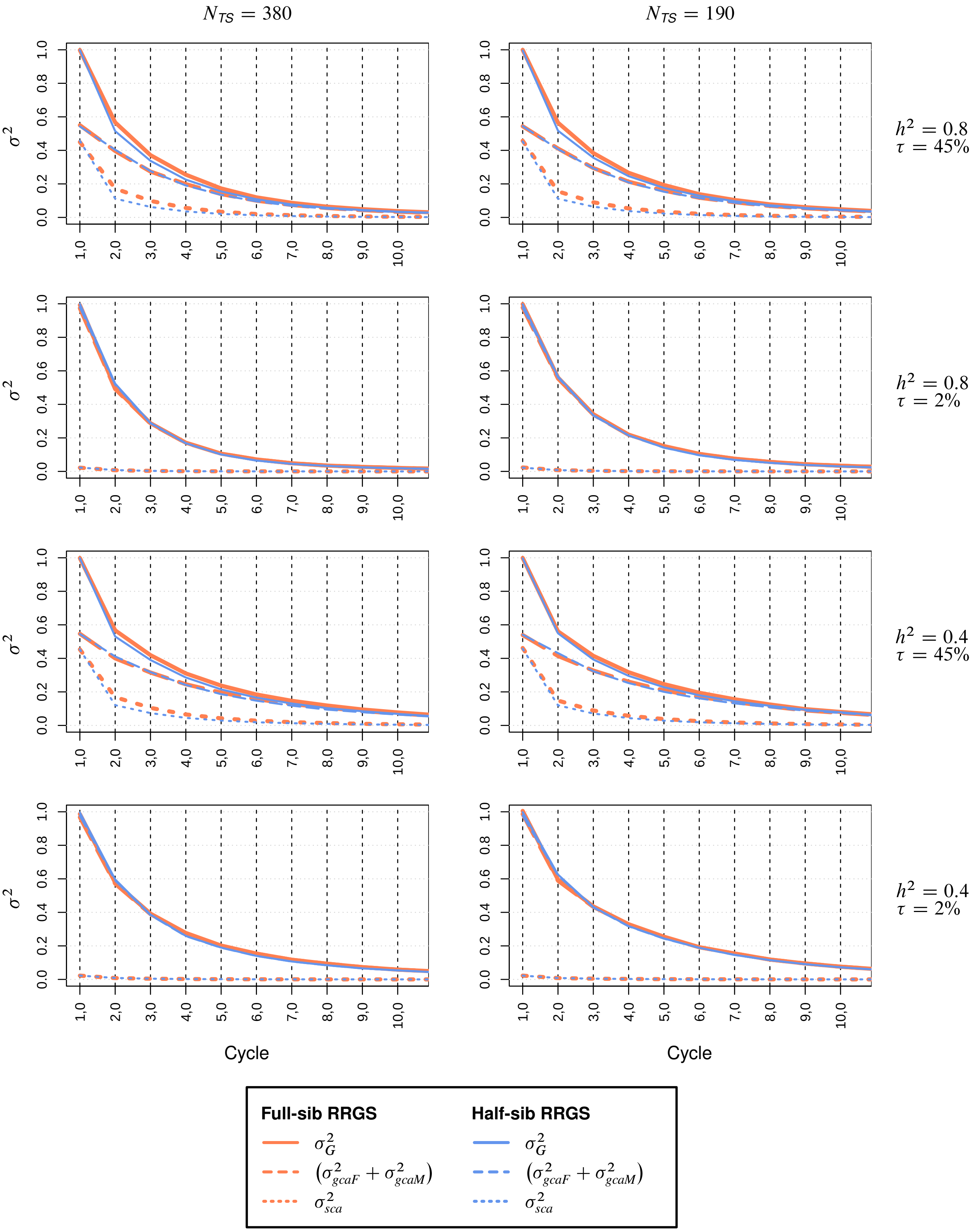

Figure S4: Genetic variances σ^2_G, GCA variances $\sigma^2_{gcaF} + \sigma^2_{gcaM}$, and SCA variances σ^2_{sca} among hybrids for full-sib and half-sib reciprocal recurrent genomic selection (RRGS). Results for 10 selection cycles based on SNP data from wheat. Scenarios differed for the training set size N_{TS}, heritability h^2, and proportion $\tau = 100\% \times \sigma^2_{sca} : \sigma^2_G$ of the trait.

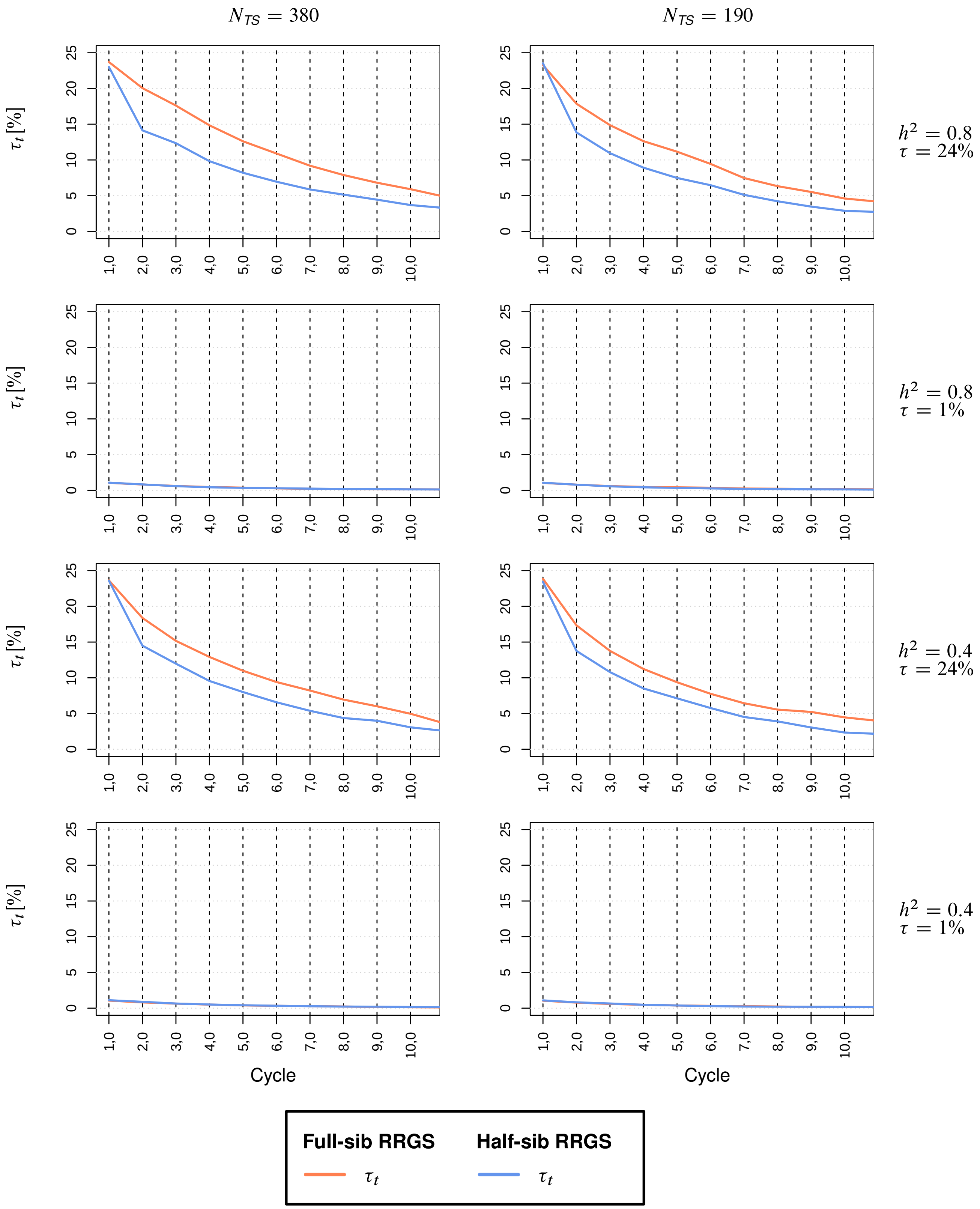

Figure S5: Proportion $\tau_t = 100\% \times \sigma^2_{sca} : \sigma^2_G$ for full-sib and half-sib reciprocal recurrent genomic selection (RRGS). Results for 10 selection cycles based on SNP data from maize. Scenarios differed for the training set size N_{TS}, heritability h^2, and proportion $\tau = 100\% \times \sigma^2_{sca} : \sigma^2_G$ of the trait.

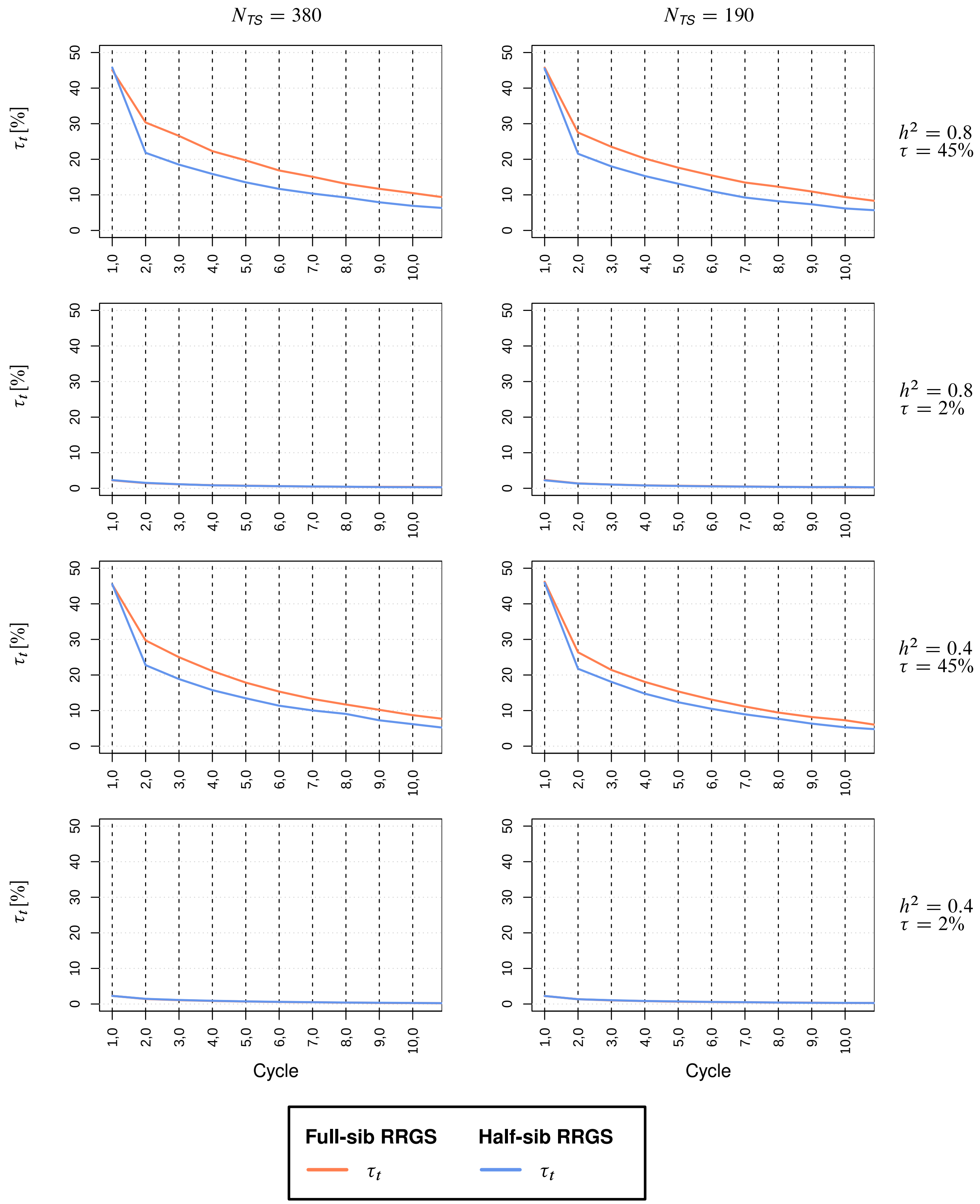

Figure S6: Proportion $\tau_t = 100\% \times \sigma^2_{sca} : \sigma^2_G$ for full-sib and half-sib reciprocal recurrent genomic selection (RRGS). Results for 10 selection cycles based on SNP data from wheat. Scenarios differed for the training set size N_{TS}, heritability h^2, and proportion $\tau = 100\% \times \sigma^2_{sca} : \sigma^2_G$ of the trait.

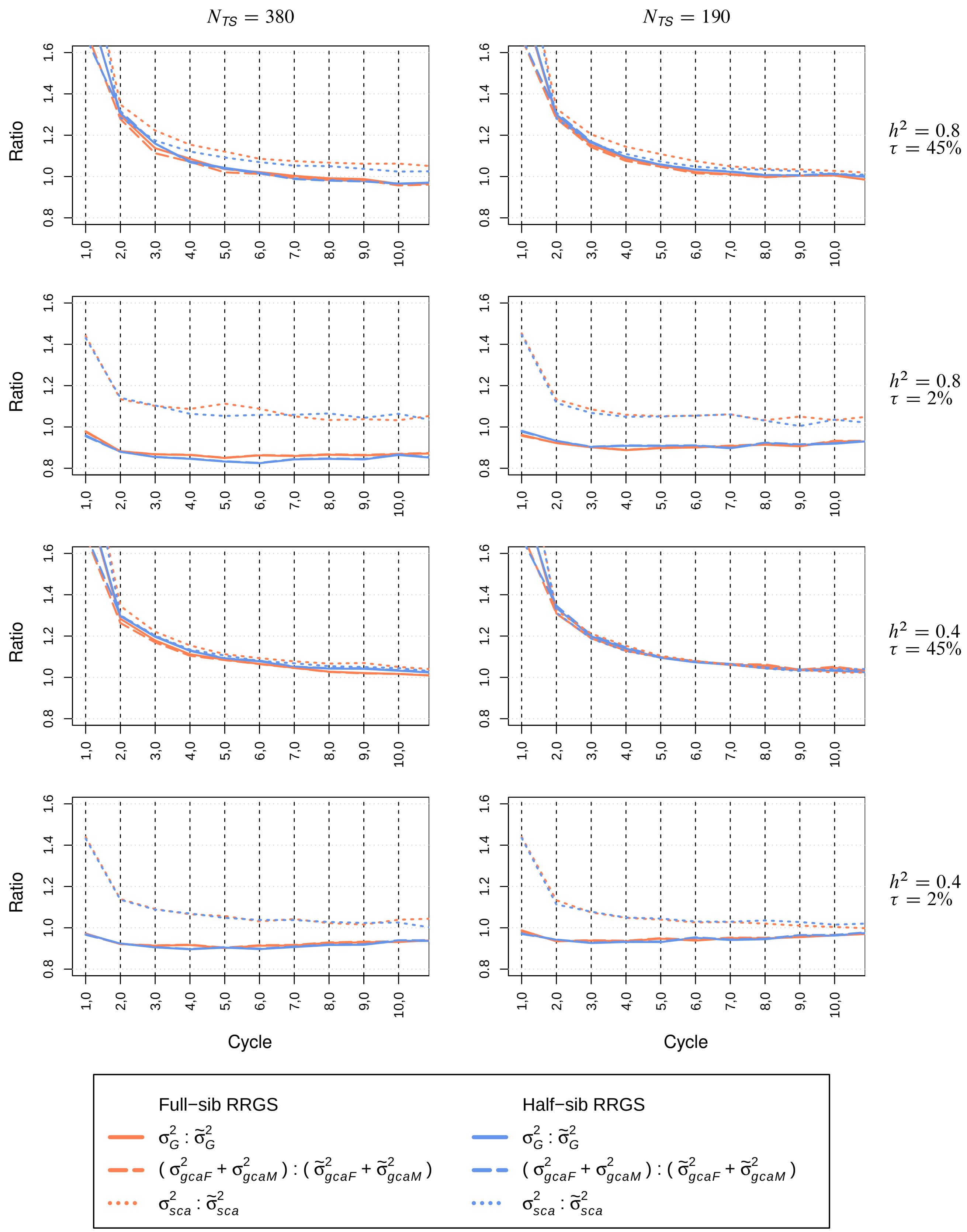

Figure S7: Ratio of genetic to genic variances $\sigma^2_G : \tilde{\sigma}^2_G$, of genetic to genic GCA variances $\left(\sigma^2_{gcaF} + \sigma^2_{gcaM}\right) : \left(\tilde{\sigma}^2_{gcaF} + \tilde{\sigma}^2_{gcaM}\right)$, and genetic to genic SCA variances $\sigma^2_{sca} : \tilde{\sigma}^2_{sca}$ among hybrids for full-sib and half-sib reciprocal recurrent genomic selection (RRGS). Results for 10 selection cycles based on SNP data from wheat. Scenarios differed for the training set size N_{TS}, heritability h^2, and proportion $\tau = 100\% \times \sigma^2_{sca} : \sigma^2_G$ of the trait.

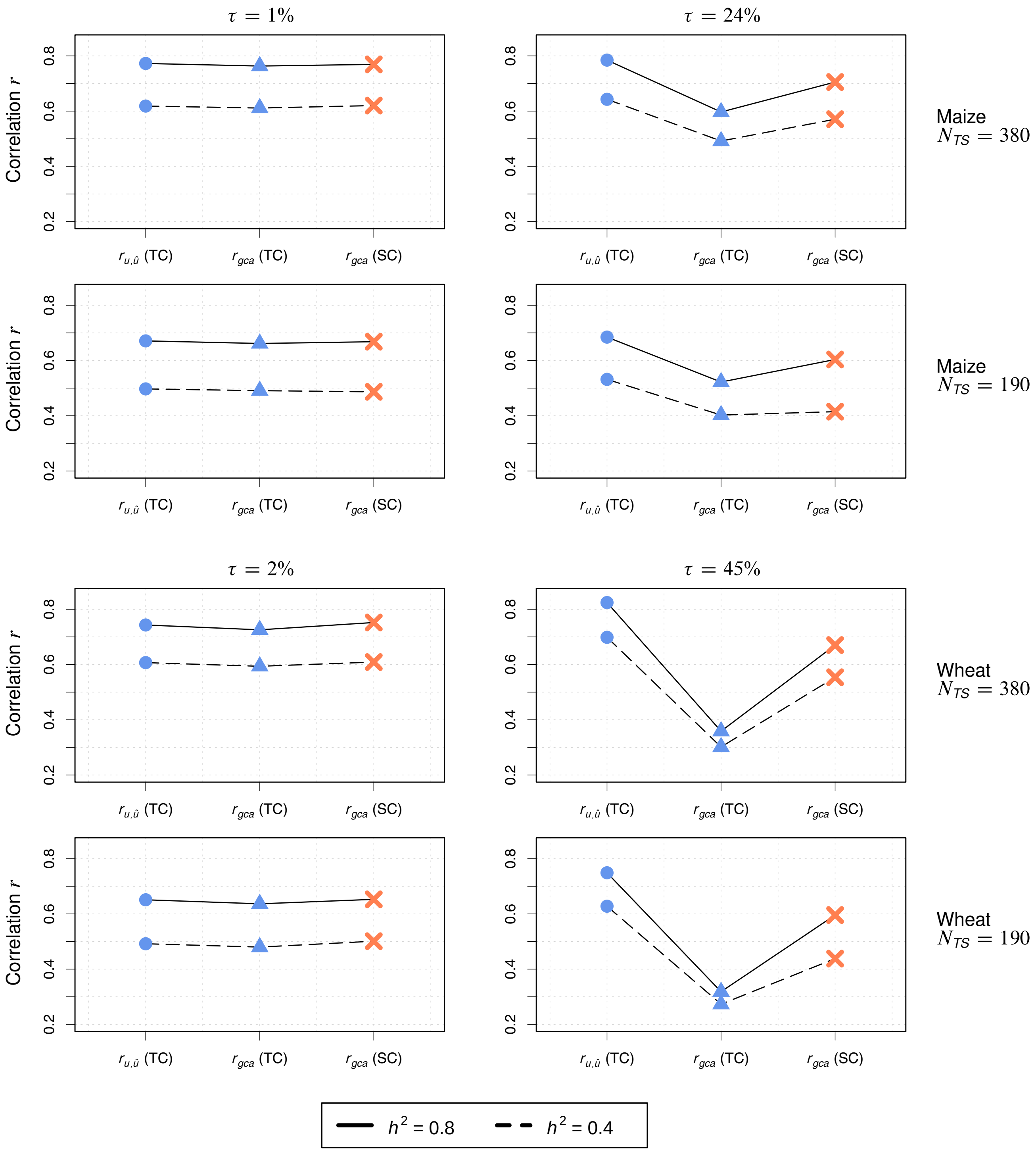

Figure S8: Prediction accuracy for testcross performance $r_{u,\hat{u}}$(TC) and for GCA r_{gca}(TC) in half-sib reciprocal recurrent genomic selection, and for GCA in full-sib reciprocal recurrent genomic selection r_{gca}(SC). Results for selection cycle $C_{1,0}$. Scenarios differed for the training set size N_{TS}, heritability h^2, proportion $\tau = 100\% \times \sigma^2_{sca} : \sigma^2_G$ of the trait, and the crop from which the SNP data were used for the simulations.

References

Beukert U, Li Z, Liu G, Zhao Y, Ramachandra N, Mirdita V, Pita F, Pillen K, Reif JC (2017) Genome-based identification of heterotic patterns in rice. Rice 10:1-10

Fischer S, Maurer HP, Würschum T, Möhring J, Piepho H-P, Schön C-C, Thiemt EM, Dhillon BS, Weissmann EA, Melchinger AE, Reif JC (2010) Development of heterotic groups in triticale. Crop Science 50:584-590

Gowda M, Longin CFH, Lein V, Reif JC (2012) Relevance of specific versus general combining ability in winter wheat. Crop science 52:2494-2500

Gramaje LV, Caguiat JD, Enriquez JOS, dela Cruz QD, Millas RA, Carampatana JE, Tabanao DAA (2020) Heterosis and combining ability analysis in CMS hybrid rice. Euphytica 216:1-22

Hu D, Zhao Y, Shen J, He X, Zhang Y, Jiang Y, Snowdon R, Meng J, Reif JC, Zou J (2021) Genome-wide prediction for hybrids between parents with distinguished difference on exotic introgressions in Brassica napus. The Crop Journal 9:1169-1178

Longin CFH, Gowda M, Mühleisen J, Ebmeyer E, Kazman E, Schachschneider R, Schacht J, Kirchhoff M, Zhao Y, Reif JC (2013) Hybrid wheat: quantitative genetic parameters and consequences for the design of breeding programs. Theoretical and applied genetics 126:2791-2801

Oettler G, Burger H, Melchinger A (2003) Heterosis and combining ability for grain yield and other agronomic traits in winter triticale. Plant breeding 122:318-321

Philipp N, Liu G, Zhao Y, He S, Spiller M, Stiewe G, Pillen K, Reif JC, Li Z (2016) Genomic prediction of barley hybrid performance. The Plant Genome 9:plantgenome2016.2002.0016

Zhao Y, Li Z, Liu G, Jiang Y, Maurer HP, Würschum T, Mock H-P, Matros A, Ebmeyer E, Schachschneider R et al. (2015) Genome-based establishment of a high-yielding heterotic pattern for hybrid wheat breeding. Proceedings of the National Academy of Sciences 112:15624-15629
